# Supplementary material for: Functional connectivity drifts during sleep as a marker of fluctuations in the level of consciousness
Source: Neurosci Conscious. 2025 Dec 27;2025(1):niaf061. doi: 10.1093/nc/niaf061 (PMC12743302; doi:10.1093/nc/niaf061)
Supplement: stage1_inPrincipleAcceptance_niaf061 [file stage1_inprincipleacceptance_niaf061.docx]

**1. Manuscript Title:** Functional connectivity drifts during sleep as a marker of fluctuations in the level of consciousness

**2. Abbreviated Title:** Functional connectivity drifts during sleep as a marker of fluctuations in the level of consciousness

**3. Authors:** João Patriota^1, 2, 3^*, Giulia Moreni^1^, Jorge Mejias^1^, Lucia Talamini^2, 3, 4^, Umberto Olcese^1, 3, 4^*

^1^ Cognitive and Systems Neuroscience Group, Swammerdam Institute for Life Sciences, University of Amsterdam, Amsterdam, The Netherlands

^2^ Brain and Cognition, Dept. of Psychology, University of Amsterdam, Amsterdam, The

Netherlands

^3^ Amsterdam Brain and Cognition, University of Amsterdam, Amsterdam, The Netherlands

^4^ Senior author

**4. Author Contributions**: Conceptualization: JP, UO; Data curation: JP; Formal analysis: JP; Funding acquisition: UO, LT; Investigation: JP; Methodology: UO, JP, GM, JM; Project administration: UO, JP; Resources: UO, LT; Software: JP, GM; Supervision: UO; Validation: UO, LT, JM; Visualization: JP, UO; Writing – original draft: JP, UO, GM, JM; Writing – review & editing: JP, UO;

#

# **Abstract**

During the wake-sleep cycle, consciousness waxes and wanes, and this is thought to be reflected in varying levels of integration between brain areas. Recent studies challenged the notion that consciousness is homogeneously present or absent in a brain state, as exemplified by conscious reports found in otherwise unconscious Non-REM sleep. We will test if functional connectivity between neurons varies within brain states in a way compatible with a fluctuating level of consciousness. We will examine directed functional connectivity between neurons across the wake-sleep cycle in rats, at a scale of a few seconds. We will analyze patterns of functional connectivity to determine if Non-REM sleep contains epochs in which inter-areal integration is comparable to that observed in wakefulness and REM sleep, and vice versa. This study will potentially reveal if circuit-level connectivity patterns are observed during sleep stages, in line with the presence of an alternation between levels of consciousness not only between but also within brain states.

**Introduction**

Every day consciousness fades when we fall asleep and is restored upon waking up - making sleep a key model for studying the level of consciousness [(Nir et al., 2013)](https://www.zotero.org/google-docs/?WUPlSL). However, sleep is not synonymous with a complete absence of consciousness [(Koch et al., 2016; Siclari et al., 2017)](https://www.zotero.org/google-docs/?6otBQg). Early descriptions of rapid eye movement (REM) sleep [(Aserinsky & Kleitman, 1953)](https://www.zotero.org/google-docs/?LQCnnx) discussed its association with vivid reports of conscious experiences (dreams). In contrast, Non-REM (NREM) sleep had classically been associated with the polar opposite: oblivion and complete disconnection [(Koch et al., 2016)](https://www.zotero.org/google-docs/?iMi79b) - but see [(Siclari et al., 2017)](https://www.zotero.org/google-docs/?NHcZwi). Varying consciousness levels across wakefulness, NREM, and REM sleep may be related to the brain network's functional integration [(Koch et al., 2016; Lu et al., 2012; Massimini et al., 2005; Olcese et al., 2016)](https://www.zotero.org/google-docs/?oEU3t5), as has also been hypothesized by leading theories of consciousness [(Storm et al., 2024; Tagliazucchi et al., 2013; Tononi et al., 2024)](https://www.zotero.org/google-docs/?WH65VC). In line with this, evidence suggests that the network transitions from globally integrated in wakefulness to disintegrated during NREM, but not REM sleep [(Massimini et al., 2005; Spoormaker et al., 2012)](https://www.zotero.org/google-docs/?Mc6JGD). These studies generally considered sleep stages as homogeneous, and thus quantified the average levels of integration separately per brain state.

However, sleep stages can be highly heterogeneous. First, slow wave activity (SWA) is now understood to be a spatially restricted phenomenon, as indicated by its local nature [(Vyazovskiy et al., 2011)](https://www.zotero.org/google-docs/?Kkpmh3). Therefore, during NREM sleep different brain regions can exhibit distinct activity profiles. This includes phenomena such as local sleep and wakefulness [(D’Ambrosio et al., 2019; Huber et al., 2004; Nobili, 2012; Vyazovskiy et al., 2011)](https://www.zotero.org/google-docs/?FmfMOS). An analogously heterogeneous picture has emerged for what pertains to the presence or absence of consciousness during sleep. For example, dream reports were thought to be exclusive to REM sleep, but consistent accounts of unconscious periods during REM sleep and conscious experiences during NREM sleep challenge this idea [(Juan et al., 2023; Siclari et al., 2017)](https://www.zotero.org/google-docs/?U0TmJq). Importantly, variations in EEG oscillatory dynamics within NREM and REM sleep correlate with dream reports, implying a dependency of dreams in local processes. Specifically, dream reports in both NREM and REM were found in a high-density EEG study to correlate with a decrease in slow wave activity and an increase in high-frequency oscillations in the posterior cortex [(Siclari et al., 2017)](https://www.zotero.org/google-docs/?RultuQ). This suggests that, even within brain states traditionally associated with the absence or presence of consciousness (NREM and REM sleep, respectively), spatiotemporally localized patterns of activity can emerge and potentially support conscious mentation.

Moreover, sleep hosts essential processes for physical and mental functions. For instance, sleep’s crucial role in memory consolidation [(Rasch & Born, 2013)](https://www.zotero.org/google-docs/?aXnIey) is reflected in coordinated reactivation of activity between the hippocampus and cortical regions during NREM sleep [(Chen & Wilson, 2023; Ji & Wilson, 2007; Sanders et al., 2019)](https://www.zotero.org/google-docs/?rbBqtP), when most of the cortical network appears to be disconnected in comparison to wakefulness [(Massimini et al., 2005)](https://www.zotero.org/google-docs/?A5wexp). Motor learning has long been known to locally modulate the amplitude of slow wave activity during NREM sleep: enhanced low power oscillations are observed in the motor cortex after learning a new motor task [(Huber et al., 2004)](https://www.zotero.org/google-docs/?gAP8Lv), and the opposite occurs after arm immobilization [(Huber et al., 2006)](https://www.zotero.org/google-docs/?XMn7PC). Importantly, a causal link was found between increased slow wave activity and memory consolidation [(Marshall et al., 2006)](https://www.zotero.org/google-docs/?VuhbMg). Experience-dependent plasticity has also been shown to enhance coordinated activity between distal areas, as exemplified by the selective synchronous reactivation of memory traces occurring in connected hippocampal and cortical ensembles REF. Along this line, we previously showed that transitions between brain states do not homogeneously modulate functional connectivity (FC) [(Olcese et al., 2016, 2018)](https://www.zotero.org/google-docs/?gVJ0hF). Specifically, intra-areal FC is generally preserved between wakefulness and NREM sleep. Conversely, inter-areal FC appears mostly depressed in NREM compared to wakefulness, although communication between hippocampus and cortex is generally preserved, and coupling between neurons significantly modulated during task performance in wakefulness can even be enhanced [(Olcese et al., 2016, 2018)](https://www.zotero.org/google-docs/?pMhr3d). However, no study so far - to our knowledge - has investigated the question of whether FC only varies between brain states or also - similar to what occurs for neural activity - also within brain states.

The diversity in the forms of neural activity brain activity that has been observed within brain states has led us to hypothesize that the functional architecture present across the brain network might also not be homogeneous within individual brain states. Specifically, while a global decrease in FC during NREM sleep compared to both REM and wakefulness is expected, different types of activity occurring in specific periods of time or subnetworks might lead to distinct levels of integration - and thus consciousness. Here we hypothesize that FC observed in a given time point might deviate in terms of both level of integration and specific connectivity patterns from what is on average observed during a brain state.

We will specifically study whether the FC structure (the set of FC values between individually recorded neurons) within NREM and REM sleep is either homogeneous or not within single brain states. To address this question, we recorded single-unit spiking activity from four brain regions of freely moving rats across wakefulness and sleep. To quantify the FC between individual neurons of different regions across sleep stages we will utilize a recently developed method called Current-Based Decomposition (CURBD) [(Perich et al., 2020)](https://www.zotero.org/google-docs/?ZSOqzA).

Specifically, we hypothesize:

**Hypothesis 1 (H1):** A significant fraction of NREM epochs display a structure of inter-areal FC that is indistinguishable from what is observed on average in REM and/or wakefulness. **Null hypothesis (H0)**: NREM epochs generally exhibit a lower level of FC in comparison to REM and wakefulness or, for those NREM epochs with average FC values comparable to those observed in REM and/or wakefulness, a structure that is different from that observed in REM and wakefulness.

**Hypothesis 2 (H2):** A significant fraction of REM epochs display a structure of inter-areal FC that is indistinguishable from what is observed on average in NREM. **H0**: REM epochs generally exhibit a higher level of FC in comparison to NREM or, for those REM epochs with average FC values comparable to those observed in NREM, a structure that is different from that observed in NREM.

This study investigates sleep-stage-specific neural dynamics, by quantifying how FC changes within and between cortical and subcortical areas across brain states. This will deepen our understanding of the nuanced neural dynamics across different sleep stages, potentially shedding light on how such patterns impact cognitive processing and consciousness.

**Methods**

In the present study, we will utilize a dataset that was originally acquired for a different research project and to answer a separate research question. Specifically, the current study will use a portion of the previously collected data that has not yet been used for any analysis. Therefore, the current study will constitute an instance of data re-use. All methods employed to record this dataset will be discussed below, as well as details on how this research question can be answered with this dataset and on considerations about the sample size. Being a data re-use project, we will only be able to reach conclusions if the previously collected dataset provides sufficient statistical power.

The Methods section is structured into five sections. The section “Experimental paradigm” describes the procedures used to collect the data. The section “Analysis plan” focuses on all analytical procedures, from pre-processing to CURBD model fitting. The section “CURBD validation” describes a preliminary analysis that we performed on simulated data to validate the application of the CURBD model and determine the minimal number of neurons for which the approach returns valid results. The section “Statistical plan” describes the hypotheses in depth, how we plan to address them and all the necessary statistical analyses that we have planned. Finally, the results of some pilots analysis that we performed to test the proposed methodology are presented in the “Pilot Analysis” section.

**Experimental paradigm**

**Ethical approval**

All experiments were performed in accordance with the National Guidelines on Animal Experiments and were approved by the National Animal Experimentation Committee and by the Animal Welfare Committee of the University of Amsterdam. Additionally, we complied with the ARRIVE guidelines [(Percie du Sert et al., 2020)](https://www.zotero.org/google-docs/?MyJq6D)

## **General Task Design**

Our study employed a within-subject five-day fear conditioning and extinction task. Over the course of five days, we sequentially carried out a series of habituation, fear conditioning, and fear extinction tasks. In this study we will utilize a portion of the original dataset, comprising the first three days of experiments (habituation, fear conditioning and fear test). During this period multi-area tetrode recordings were performed across both task performing and rest sessions. The fear conditioning and extinction task is not directly relevant for the purposes of the present study. Specifically, as described more in depth later (section “Experimental procedure”), we expect that any consequence of fear conditioning or extinction on neural activity and connectivity will not affect our research question of whether the FC structure is homogeneous or not within individual brain states. For this reason, we believe that the re-use of an available dataset to answer an independent research question is more ethically responsible compared to performing an ex novo experiment. For the sake of completion, we will nonetheless provide details about the complete experimental paradigm that was performed, even when not directly relevant to our research question.

**Experimental animals**

Three male Lister Hooded rats (aged 2-5 months, Envigo, The Netherlands) were housed in pairs during behavioral training. Following the implantation of a tetrode drive, rats were individually housed in transparent cages (53x53x60 cm). All rats were maintained on a regular (i.e., non-reverse) 12-h light/12-h dark schedule and tested in the light (inactive) period. During all experiments rats were fed in an ad libitum regime. After about 5-6 hours of recordings the animals were placed back in their home cage in the animal facility, and allowed to sleep for the rest of the light-on period.

**Behavioral apparatus**

Behavioral measurements were performed in a plexiglass box, with an electric grid (equidistant spaces of 1cm.) on the bottom (Fig. 1A; width 53 (base) x length 53 x height 60 cm). Current delivery was controlled by a dedicated scrambled grid current generator (MANHSCK100A, Lafayette). The transparent walls allowed us to present different visual cues via drawings placed on the walls, to further enhance the context of each behavioral stage. Sleep recordings took place in a nest (30 x 30 x 10 cm), placed in the same position in the room as the plexiglass box. Importantly, the nest was only used to allow sleep recordings, but was placed under a different context than the box. During the recordings, auditory stimuli were presented at 60dB (zylux multimedia speaker system, Dell).

**Behavioral paradigm**

In preparation for the experiment, animals were gradually acclimatized to the experimental setup over a two-week period. Each animal was familiarized daily with the experimental box by spending at least thirty minutes within it, followed by an equal amount of time spent in the nest (Fig 1A).

Upon completion of this acclimatization phase, electrode implantation followed. The behavioral paradigm was initiated at least fourteen days after surgery, to ensure full health of the animal, and lasted for up to five consecutive days. Each daily experimental session was divided into two distinct phases. In the first phase, during about 60 minutes, animals were given freedom to navigate the experimental box while engaging in the assigned task. Once this task was completed, the subjects were relocated to the nest for sleep recordings that took place for about 5h.

Detailed descriptions of each experimental day's activities are elaborated upon in the subsequent section.

**Detailed Experimental Procedure**

In this experiment, we followed an auditory cue fear conditioning paradigm [(Bagur et al., 2021; LeDoux, 2014)](https://www.zotero.org/google-docs/?MfexWp).

**Day 1: Habituation** - The initial day of the experiment entailed habituation. Each animal was placed in the experimental box and we initiated the habituation phase. This phase involved 25 presentations of two types of auditory stimuli (Conditioned Stimuli or CS, 2s white noise filtered at 3kHz, and white noise filtered at 9kHz, respectively). Each CS presentation was interspersed with a randomized inter-trial interval (ITI) of 3 to 5 minutes. The auditory stimuli were presented in a randomized sequence.

**Day 2: Fear Conditioning** - On the second day, each animal was placed in the experimental box. Visual cues were added to enrich the context (Fig. 1A). Animals were then subjected to fear conditioning procedures. Post baseline period (3 minutes), the animals were introduced to 10 trials comprising five CS- and five paired CS+-Unconditioned Stimulus (US) presentations [(Bagur et al., 2021)](https://www.zotero.org/google-docs/?B6gRiE). The same CS stimuli used in day one were presented, with one stimulus being used as CS+, and the other as CS-. The US, a foot shock (500ms, 0.5mA), was delivered immediately following the end of the sound. The ITI was randomized between 3 to 5 minutes. Each sound lasted 2 seconds, with the shock administered instantly after the sound via a specified controller.

**Day 3: Fear Test** - On the third day, the probe test was conducted. Animals were placed in the experimental box, visual aid on the plexiglass walls were the same used during the fear conditioning step. Following the baseline period (3 minutes), the animals were presented with 12 trials including five CS- and seven CS+ pairs [(Bagur et al., 2021)](https://www.zotero.org/google-docs/?dyt1o5). Sound presentation was conducted in a block format: initially, all CS+ sounds were presented, followed by a block of CS- sounds. The ITI was again randomized between 3 to 5 minutes, with each sound lasting 2 seconds.

Subsequent experimental procedures, unrelated to the primary focus of this study, spanned another two days, after which the animal was euthanized.

**Day 4: Extinction training -** On the fourth day, each animal was placed in the experimental box and we initiated the extinction phase. Here the visual cues were different from the ones used during days two and three. This phase involved 30 presentations of both CS+ and CS-. Each CS presentation was interspersed with a randomized inter-trial interval (ITI) of 3 to 5 minutes. The auditory stimuli were presented in a randomized sequence.

**Day 5: Extinction test** - On the fifth day, the extinction test was conducted. Following the baseline period, the animals were presented with 12 trials including five CS- and seven CS+ pairs [(Bagur et al., 2021)](https://www.zotero.org/google-docs/?wZT4yM). Sound presentation was conducted in a block format: initially, all CS+ sounds were presented, followed by a block of CS- sounds. The ITI was again randomized between 3 to 5 minutes, with each sound lasting 2 seconds.

Here we will combine data recorded across different experimental conditions, under the assumption that brain states will similarly modulate inter-areal communication. In other words, we expect that the homogeneous or heterogeneous nature of the FC structure within individual brain states will not depend on the experimental phase. However, it is likely that each experimental condition might differentially affect how brain states modulated functional coupling between brain regions (i.e., the details of the FC structure itself). Previous literature suggests that this effect would be heterogeneous, and would differentially impact not only different brain areas, but also specific neuronal subpopulations within areas - see e.g.[(Ji & Wilson, 2007; Lansink et al., 2009; Olcese et al., 2016)](https://www.zotero.org/google-docs/?BV8UMP). Based on the results that we will obtain, this might be a topic for further investigation.

**Sleep Recordings** - Sleep sessions were conducted immediately after each behavioral session. Animals were relocated to a dimly lit flower pot for sleep recordings, each session lasting approximately five hours. Upon identifying sleep onset, a period defined by the emergence of significant slow-wave activity (SWA) in the cortical signals, coupled with a notable decrease in the animal's motion (see also later), as detected via our camera-based monitoring system, both CS+ and CS- stimuli were randomly played, supplemented by a novel sound (white noise filtered at 6kHz, 2s). Inter stimulus interval was randomly set between 2 and 4 seconds. If an arousal was observed by the experimenter, sound presentation ceased immediately. Arousals were identified as any significant disruptions in ongoing stereotypical wave patterns, such as theta waves during REM sleep or delta waves during NREM sleep. Additionally, these disruptions were concurrently verified through motion observations, providing a multimodal approach to effectively detect instances of arousal in the animals. Each session featured approximately 200 sound presentations.

**Electrode implantation and surgery**

In order to allow full recovery and to avoid interferences on the experimental procedures, surgery was performed at least 14 days prior to the first experimental day (habituation). Tetrodes were constructed from four twisted 13-µm coated nichrome wires (California Fine Wire). The electrode tips were gold-plated to reduce electrode impedances to 300-800 kΩ at 1 kHz. Tetrodes were loaded into a custom-built microdrive, containing 36 individually movable tetrodes (Bos et al., 2017; Lansink et al., 2007). The drives targeted eight tetrodes to the primary auditory cortex (A1; target coordinates in mm: -4.81 AP, -3.81 ML, 4.32 DV (Paxinos and Watson, 2007)), eight tetrodes to dorsal hippocampal CA1 area (-3.2 AP, -1.51 ML, 2.88 DV), eight tetrodes to the basolateral amygdala (BLA; -2.74 AP, -3.35 ML, 7.2 DV) and eight tetrodes to the prefrontal cortex (mPFC; +1.83 AP, -0.83 ML, 5.33 DV).

Prior to surgery, rats received a subcutaneous injection of buprenorphine (Buprecare, 0.04 mg kg-1), meloxicam (Metacam, 2 mg kg-1) and enrofloxacin (Baytril, 5 mg kg-1). Anesthesia was induced by placing the animal in a closed plexiglass box filled with isoflurane vapor (3.0%). During surgery the animals were mounted in a stereotaxic frame where anesthesia was maintained using isoflurane (1.0-2.0%) and body temperature was maintained between 35 and 36 deg using a heating pad. Local anesthetic (lidocaine) was applied directly on the periosteum before exposing the skull. The skull was thoroughly cleaned with a 3% hydrogen peroxide solution to roughen the skull surface. The hydrogen peroxide was removed by rinsing the skull three times using saline. Six screws, from which one in the occipital bone served as ground, were inserted into the skull to improve the stability of the implant. After craniotomy and durotomy were performed, the drive was positioned over the craniotomies. The craniotomies were sealed using silicone adhesive (Kwik-Sil), the skull was covered with a layer of dental adhesive (OptiBond, Kerr) and primer (3M Transbond) and finally the drive was anchored using dental cement (Kemdent). Finally, all tetrodes were lowered into the superficial layers of the cortex. From the third day, tetrodes were gradually lowered towards their target location regions over a span of 7-14 days. Post-operative care included a subcutaneous injection of meloxicam once per day for two days following surgery, and a single injection of enrofloxacin on the day following surgery.

**Data acquisition**

For data collection, the rat was connected to the recording equipment (Open Ephys, 148 channels) via operational amplifiers (RHD2000, Intan) and a tether cable connected to a commutator (Saturn-5, Neuralynx). After the post-surgery recovery period, tetrodes were lowered to their target location while their current location in the brain was estimated based on the distance the tetrode was moved in the drive and through thorough assessment of the LFP and spike signals. In order to sample activity from a diverse population of neurons, tetrodes were independently advanced after each recording session by at least 250 um. Each tetrode was moved independently to a location where spiking activity was clearly visible in the raw trace. During experiments the neural activity was acquired at 30 kHz continuously and stored on a hard drive for further processing. A camera (acA1920-25gm, Basler) positioned above the behavioral setup recorded behavioral activity. The behavioral apparatus was controlled using an Arduino Mega (Arduino). Programmed commands and events recorded were sent to the Open Ephys recording system as TTL pulses. Animals were equipped with a headstage (Intan RHD2132 board) which includes a 3-axis accelerometer that gives access to the animals’ acceleration with high temporal resolution.

**Histology**

After the final recording session, currents (12 µA, 10 s) were applied to one lead of each tetrode to mark its endpoint with a small lesion. Twenty-four hours after lesioning, the animals were deeply anesthetized with Pentobarbital (Nembutal, Ceva Sante Animale, 60 mg ml-1, 1.0 ml intraperitoneal) and transcardially perfused with saline followed by a 4% paraformaldehyde solution (pH 7.4, phosphate buffered). After a minimum of 24h post-fixation, the brain was sectioned into 40-50µm thick slices using a vibratome. A Nissl staining (Cresyl Violet) was set to mark cell bodies and to reconstruct tetrode tracks and localize their endpoints. The sections were imaged and aligned to the 3D Waxholm reference atlas (Papp et al., 2014)

## **Analysis Plan**

**Motion tracking**

Animal recordings were equipped with a headstage (Intan RHD2132 board) which included a 3-axis accelerometer which gave access to the animal’s acceleration with high temporal resolution. This information was used to measure motor activity in the same temporal resolution as the collected local field potential activity [(Bagur et al., 2021)](https://www.zotero.org/google-docs/?r0P5iP).

**Spike detection**

Signals were digitally band-pass filtered between 800 and 6000 Hz for spike recordings. Action potentials were assigned to single neurons by using Tridesclous, a semiautomated spike sorting algorithm (Garcia & Pouzat, 2019) implemented within the Python package spikeinterface [(Buccino et al., 2020)](https://www.zotero.org/google-docs/?E45y9u), and manually curated using the Phy GUI (Rossant et al., 2016). Each candidate unit was considered for further inspection during manual curation based on its waveform and autocorrelation function. High-quality single units were included, and defined as having (1) an isolation distance higher than 10, (2) less than 0.1% of their spikes within the refractory period of 1.5 ms, (3) spiking activity present throughout the session. Other candidate units, with physiological waveforms but with more than 0.1% of their spikes within the refractory period (i.e., still respecting criteria number 1 and 3), were deemed as high-quality multi units and were included for the CURBD analysis. Local field potentials (LFPs) were recorded from all tetrodes, continuously sampled at 30 kHz, and band-pass filtered between 1 and 1000 Hz. Across 7 sessions, we obtained on average, per area (mean ± standard deviation): 6.3 ± 1.4 in BLA, 6.6 ± 3.1 in PFC, 5.9 ± 4.64 in A1, 3.43 ± 1.29 in HPC (Table 5). For further description of the dataset, see the sampling plan below.

**Sleep scoring**

Considering the fragmented nature of rodent sleep, we segmented the recording sessions into discrete 5-second epochs [(Olcese et al., 2018)](https://www.zotero.org/google-docs/?HQpkrQ), individually scored for analysis (Fig. 1B). This scoring was done by simultaneously evaluating several factors: cortical Local Field Potential (LFP) channels, LFP power spectrum within each epoch and motor activity (ascertained through accelerometer displacement). More in detail, sleep stage scoring was done by taking into account LFP oscillatory dynamics in each epoch of multiple cortical recording channels as well as the level of motor activity (measured in terms of head acceleration). Epochs that displayed 1) low levels of cortical slow-wave activity (total power between 0.5 and 4 Hz) and detectable levels of motor activity (head acceleration over a visually determined threshold of 750 arbitrary units - see Fig 1C) or 2) no detectable motor activity in said epoch, but with motor activity above the threshold in the previous 10s (two epochs) or subsequent 10s were classified as wakefulness (WAKE). The motor threshold was determined by measuring head acceleration during periods when video recordings showed freezing or immobility in the absence of motor actions such as grooming [(dos Santos Lima et al., 2019)](https://www.zotero.org/google-docs/?Gf1Xw3). We also classified an epoch as WAKE if, on top of the lack of movement, we did not observe theta oscillations. The presence of theta oscillations (in the absence of motion) was instead taken as an indication of REM sleep. Epochs with a high level of slow-wave activity and limited motion were scored as NREM. Overall, this evaluation allowed us to classify the behavioral state of recorded animals into three distinct stages: wakefulness (WAKE), NREM sleep and REM sleep. In case the typical markers of individual sleep stages were absent or were not consistently present in all recorded areas, we classified such epochs as undefined (UND). This was for example the case in the transition between sleep stages, when epochs can show intermediate patterns of activity either temporally (within each epoch) or spatially (across areas) [(Emrick et al., 2016)](https://www.zotero.org/google-docs/?LBrYFO). Such epochs were excluded from further analysis. Importantly, this exclusion criterion makes it unlikely that, even in the absence of EEG and EMG recordings, we will spuriously include local brain states [(Vyazovskiy et al., 2011)](https://www.zotero.org/google-docs/?QEr8ck) in our analysis. Specifically, our recording sites span from posterior areas (auditory cortex) to frontal ones (prefrontal cortex) and even deep regions (hippocampus, basolateral amygdala). Therefore, we deem it very unlikely that any (region-specific) sleep stage marker simultaneously detected in all recording sites would reflect a local rather than global phenomenon.

These stringent criteria, which include a conservative acceleration threshold to detect immobility [(Bagur et al., 2021; dos Santos Lima et al., 2019)](https://www.zotero.org/google-docs/?8hJrOi), were used to identify motor activity and to facilitate the identification of sleep stages, contributing to the validity of our results. We corroborated the outcomes of our behavioral scoring procedure by separately plotting several electrophysiological and behavioral parameters for each recording session (Fig. 1B). Hippocampal ripples, typical of quiet wakefulness and NREM but less frequent in active wakefulness, further validated the effectiveness of our scoring procedure.

Across 7 sessions we obtained, on average, per sleep stage: 2023.86 ± 523.74 NREM epochs, 779.57 ± 224.64 REM epochs and 1242.43 ± 445.31 epochs of wakefulness. For further description of the dataset, see the sampling plan below. Epochs that failed to meet the criteria for any of these four states were discarded [(Olcese et al., 2016, 2018)](https://www.zotero.org/google-docs/?W6DFaG).

We then employed a custom script to generate comprehensive information on time-frequency, power source distribution, downsampled data visualization, and animal motion. This process provided a platform to visualize the data, plot 5-second epochs, and subsequently calculate the Hilbert amplitudes (smoothed with a Gaussian kernel, σ = 1 s) of filtered theta (6–12 Hz) and delta (1–4 Hz) band LFP (Fig. 1B).

**Estimation of connectivity**

Building upon the already done preprocessing (spike sorting and sleep stage categorization), we will separate Single Unit Activity (SUA) into distinct trials for each 5 s sleep epoch. Current-Based Decomposition (CURBD) [(Perich et al., 2020)](https://www.zotero.org/google-docs/?BvNd3F) will be applied on an epoch by epoch basis to the spiking trains from different pairs of neurons belonging to different regions, in order to obtain the connectivity magnitude of all combinations per each epoch. Compared to common methods to study interactions between brain regions such as linear regression, canonical correlation analysis (CCA), constrained dimensionality reduction, generalized linear models (GLMs), or Granger causality, amongst others, CURBD aims to reconstruct underlying connectivity by reproducing the observed activity patterns, rather than just computing (directional) correlations between activity patterns. In this way, CURBD allows us to, in an unbiased way, capture the directionality and magnitude of the interactions within and across regions that can be interpreted as being computationally responsible for the observed neural dynamics (Fig. 4A). Among other strategies to fit recurrent neural networks to ensemble recordings - see e.g. [(Das & Fiete, 2020; Finkelstein et al., 2021; Kuan et al., 2024; Pandarinath et al., 2018)](https://www.zotero.org/google-docs/?k1xh4t) - CURBD stands out as it was specifically developed to infer functional connectivity between brain areas. This approach is furthermore highly valuable for our purposes, since it is valid even when applied to a low number of units [(Perich et al., 2020)](https://www.zotero.org/google-docs/?m1S29r). Importantly, this method jointly captures mono- and polysynaptic connectivity. This method thus allows to jointly quantify both direct and indirect connections between pairs of regions, providing indications about the overall strength and patterns of interareal connectivity. Since both direct and indirect connections between - but not within - brain regions are thought to be strongly affected by brain states [(El-Baba et al., 2019; Jobst et al., 2017; Olcese et al., 2016, 2018)](https://www.zotero.org/google-docs/?Jgs7fg), this method is ideally suited to determine to what extent individual brain states are homogenous in terms of multi-regional functional connectivity structures.

CURBD assumes that the high degree of recurrent connectivity present between brain regions makes a recurrent neural network (RNN) suitable to reproduce such dynamics, and that the activity that drives a unit in an RNN can be computed as a weighted sum of the activity of all other source units in the network. CURBD then fits a single layer RNN to neural activity obtained from an experiment, estimating functional connectivity with a high spatiotemporal resolution, taking in account each epoch and at the level of single neurons, but also constrained by the particularities of the experimental data in view of the multi-regional nature of our recordings. A model RNN is constructed such that each unit is trained to match a single experimentally recorded epoch from a neuron from the full dataset of neural population activity from multiple interacting regions. Importantly, the output obtained by CURBD should be considered a model of the data itself - an in silico representation of the experiment: such a model does not incorporate prior assumptions regarding the identity of the model neurons, nor does it consider anatomical constraints. Training occurs where the connectivity matrix of the model RNN is modified over time until the activity of the RNN units match the experimental data [(Perich et al., 2020)](https://www.zotero.org/google-docs/?3A6Co9). For each pair of neurons per epoch we will obtain a 5 s time series where each point corresponds to the estimated connectivity between those two neurons in that epoch. We will then average the pairwise connectivity values obtained over the whole 5 s epoch, for all pairs of neurons located in different brain regions, in order to obtain one single pairwise connectivity value per epoch (Fig 4B). Therefore, in the following analyses, we will not consider how connectivity between individual pairs of neurons varies within and between brain states, but rather how the average value of pairwise interactions between areas is modulated. Thus, we will not aim to reconstruct the connectivity diagram between the recorded neurons, but only focus on the average connection strength between regions. While connectivity will be estimated independently for each 5 s epoch, results will be analyzed first at the level of each recording session, and then combined across sessions. Details are provided in the following sections.

As discussed, CURBD was designed to infer interactions in experimental datasets. However, the method has not been yet validated in a peer-review publication. Therefore, we proceeded to perform a pilot analysis to determine whether CURBD is capable of capturing the dynamics of interareal functional connectivity in a biologically inspired spiking neural model based on previous work [(Joglekar et al., 2018; Mejias & Longtin, 2014; Moreni et al., 2023, 2024; Nunes et al., 2021; Olcese et al., 2010)](https://www.zotero.org/google-docs/?XUbWzj). Basically, this toy model allowed us to simulate realistic spiking patterns in a 4-region cortical network in which we could set, on the basis of experimental data [(Oh et al., 2014)](https://www.zotero.org/google-docs/?E1zEs3), the average level of monosynaptic connectivity between areas, and also perform current injections in one area and simulate how evoked spiking activity differentially propagates to other areas based on the underlying network connectivity. Importantly, the model was not designed to reproduce the activity of the areas from which recordings were performed. Rather, it only served as a toy model to validate the functionality of CURBD, besides what has been presented in the manuscript proposing the method. The next sections describe the model in depth.

## **CURBD validation**

## **Model architecture**

The computational model, whose architecture is depicted in Fig 2A, is composed of a total number (N_total_) of 5,000 neurons. The model consists of four areas (R1-R4), each containing 85% excitatory neurons (E) and 15% inhibitory (I) neurons [(Billeh et al., 2020)](https://www.zotero.org/google-docs/?TTM1jM). Each area thus contains 1062 excitatory cells and 188 inhibitory cells. All neurons receive background noise from the rest of the brain.

#### Single neurons

All excitatory and inhibitory cells in the network are modeled as leaky integrate-and-fire neurons. Each of the two types of cell is characterized by its own set of parameters: a resting potential V_rest_, a firing threshold V_th_, a membrane capacitance C_m_, a membrane leak conductance g_L_ and a refractory period t_ref_. The corresponding membrane time constant is t_m_= C_m_/g_L_. The membrane potential V(t) of a cell is given by:

$C_{m} \frac{dV(t)}{dt} = -g_{L}(V(t) - V_{rest}) + I_{syn}(t)$ (Eq. 1)

where I_syn_(t) represents the total synaptic current flowing in the cell.

At each time point of simulation, a neuron integrates the total incoming current I_syn_(t) to update its membrane potential V(t). When the threshold V_th_ is reached a spike is generated, followed by an instantaneous reset of the membrane potential to the resting membrane potential V_rest_. Then, for a refractory period t_ref_, the membrane potential stays at its resting value V_rest_  and no spikes can be generated. After t_ref_ has passed, the membrane potential can be updated again (see Table 1 for the corresponding parameter values).

Parameters of neuron models

Each type of cell (I or E) is characterized by its own set of parameters: a resting potential V_rest_, a firing threshold V_th_, a membrane capacitance C_m_, a membrane leak conductance g_L_ and a refractory period t_ref_.

These data are taken from the Allen institute database (<https://portal.brain-map.org/explore/models/mv1-all-layers>) of layer 2/3 of the primary visual cortex (V1), reflecting the fact that electrophysiological properties of neurons in that area have been thoroughly studied. Without loss of generality, we chose layer 2/3 as our representative subcircuit due to its particularly strong recurrent excitatory connectivity, which allowed us to design a model with a clear segregation of neurons into separate regions. We use data from pyramidal cells to model our excitatory neurons and data from PV interneurons to model our inhibitory neurons.

#### Synapses

The inputs to model neurons consist of three main components: background noise, external (e.g. sensory) input and recurrent input from the modeled areas . EPSCs due to background noise are mediated in the model by AMPA receptors (I_ext,AMPA_(t)) and EPSCs due to external stimuli (i.e. originating from outside the area) are represented by I_ext_(t). The recurrent input from within the model is given by the sum of I_AMPA_(t) and I_GABA_(t). These are all the inputs from all the other presynaptic neurons projecting to the neuron under consideration.

The total synaptic current that each neuron receives is given by:

$I_{syn}(t) = I_{ext}(t) + I_{ext, AMPA(t)} + I_{AMPA(t)}+ I_{GABA(t)}$ (Eq. 2)

with the first term on the right hand (I_ext_) side being the current injected externally (a constant current of 30 pA whenever the network receives external input, whose value was empirically determined to achieve regular model dynamics), and with the last three terms given by

$I_{ext, AMPA(t)} = g_{AMPA}(V(t) - V_{E}{)s_{ext,AMPA(t)}}$ (Eq. 3)

$I_{AMPA(t)} = g_{AMPA}(V(t) - V_{E}{)\sum_{j=i}^{N} w_{j}s_{j}AMPA(t)}$ (Eq. 4)

$I_{GABA(t)} = g_{GABA}(V(t) - V_{I}{)\sum_{j=i}^{N} w_{j}s_{j}GABA(t)}$ (Eq. 5)

where the reversal potentials are V_E_=0 mV, V_I_ = V_rest_. The g terms represent the conductances of the specific receptor types, and are equal to one for simplicity. The weights w_j_ represent the strength of each synapse received by the neuron. The sum runs over all presynaptic neurons j projecting to the neuron under consideration. The s terms represent the gating variables, or fraction of open channels and their behavior is governed by the following equations.t

The AMPAR channels are described by

$\frac{ds_{j}AMPA(t)}{dt} =\frac{-s_{j}AMPA(t)}{\tau_{AMPA}} +\sum_{k} \delta(t - t_{j}k)$ (Eq. 6)

where the time constant of the AMPA currents is t_AMPA_= 2 ms [(Wang, 2002)](https://www.zotero.org/google-docs/?KEcz8W) - see also [(Hestrin et al., 1990; Spruston et al., 1995)](https://www.zotero.org/google-docs/?Xw3axV) - and the sum over k represents the contribution of all spikes (indicated by delta,
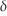
) emitted by presynaptic neuron j. In the case of external AMPA currents (Eq. 3), the spikes are emitted accordingly to a Poisson process with rate u_bkgnd_. The two groups of cells in each area (I and E) are receiving a different Poisson rate of background noise: u_bkgnd_ = 930 Hz for excitatory cells and u_bkgnd_ = 1460 Hz for inhibitory cells. These values were empirically determined, in line with what is commonly done for similar models [(Potjans & Diesmann, 2014; Van Albada et al., 2015)](https://www.zotero.org/google-docs/?Q0070x). This is roughly equivalent to having roughly ~1000 external neurons projecting to each neuron in either group and firing at around 1 and 1.5 spikes/s respectively, which constitutes a good approximation for spontaneous activity levels in cortex

The GABA_A_ receptor synaptic variable is described by

$\frac{ds_{j}GABA(t)}{dt} =\frac{-s_{j}GABA(t)}{\tau_{GABA}} +\sum_{k} \delta(t - t_{j}k)$ (Eq. 7)

where the time constant of GABA_A_ receptor current is 5 ms [(Wang, 2002)](https://www.zotero.org/google-docs/?BbhGkJ) - see also [(Salin & Prince, 1996; Xiang et al., 1998)](https://www.zotero.org/google-docs/?AiHbHV).

Connectivity and synaptic weights

Each neuron in the network projects a synapse to any other neuron with a certain probability which depends on the cell group (i.e. excitatory or inhibitory) and area (i.e. R1, R2…). The values in matrix P (Table 2) indicate the probability that a neuron in group A (e.g. an inhibitory cell in area R1) is connected to a neuron in group B (e.g. excitatory cell in area R1).

The weight of each existing synapse w_j_ from neurons of group A to neurons in group B in the same area is chosen to be equal to

$w_{j} =G\frac{ŝ}{N_{send}p}$ (Eq. 8)

where G=5 is the global coupling factor (this value was empirically chosen to guarantee a minimum level of strength of the overall connectivity without leading to pathological synchrony in the dynamics),
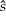
 is the overall strength between the two connected groups of cells, N_send_ is the number of neurons in the sending population A, and p the probability of connection between the neurons of the two groups (A and B) taken from the connection probability matrix (Table 2),
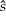
 is from the experimental synaptic connectivity matrix (Table 3). As for the other model parameters, the values are chosen from layer 2/3 of the cortical column in V1 [(Billeh et al., 2020)](https://www.zotero.org/google-docs/?JwPt0u). Complete matrices for the values of
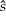
 and p of the cortical column where these values were taken from can be found at [https://portal.brain-map.org/explore/models/mv1-all-layers.](https://portal.brain-map.org/explore/models/mv1-all-layers.%20) The normalization in Eq. 8 above guarantees that the dynamics and equilibrium points of the system scale properly with the size of the network.

Here we use the term connection with reference to subpopulations or groups, defined by the pre- and postsynaptic neuron types in each area (I and E cells). The connection probability defines the probability for each possible pair of pre- and postsynaptic neurons to form a connection between them. If p=0.1 this connects all neuron pairs of the two groups with a probability of 10%. The connectivity probability matrix P within one area is defined by the 2x2 = 4 connection probabilities p between the 2 considered cell groups (E and I cells). For simplicity, and without loss of generality for our method, the same values are used for all the 4 areas. Then the four areas are connected only through excitatory neurons (see Fig. 2A) with a fixed probability p=0.3 (empirically determined).

Each connection also has a particular strength which differs per neuron group. Thus, the strength was specified at the level of neuron type X projecting to neuron type Y (Table 3). These values are rescaled to find the single synaptic strength w_j_ between neurons to use in the model (Eq. 8).

As mentioned earlier, the four areas R1- R4 are connected only through pyramidal cells (See Fig. 2A). The connections strength between excitatory cells (E) of the four different areas are taken from experimental data [(Oh et al., 2014)](https://www.zotero.org/google-docs/?A8UvRt) and are the values between four brain areas (Primary visual area, Anterolateral visual area, Barrel field in the primary somatosensory area, Dorsal part of the anterior cingulate area) which are represented by area R1-R4 in our model. We chose these areas to have a spatially distributed network including both sensory and association areas, but other areas can be easily considered as well. The values of the connection strength of single synapse w_j_ are reported in the following table (Table 4). Note how the connectivity between R1 and R2 is one order of magnitude higher than that between all other areas, to test if CURBD can capture this enhanced connectivity. These values are then increased by a global constant G=50, selected empirically to obtain realistic firing rates in all 4 areas.

Simulation details

All simulations were performed using custom Python code and Brian 2.0. Differential equations were solved using an Euler-Mayurama algorithm with an integration step of 0.1 ms. The code will be made available via GitHub upon acceptance of the manuscript.

## **CURBD validation on simulated data**

The chosen model implementation allowed us to explore if CURBD could, on the basis of the activity patterns observed in different regions, capture the predetermined inte-areal connectivity values. In this implementation, we selected connection strength between regions that are similar in magnitude from what is obtained via experimental data [(Oh et al., 2014)](https://www.zotero.org/google-docs/?GmumPj). Here, the pair R1-R2 is more strongly connected than other pairs such as R3-R4, R1-R3, etc. (Fig 2A), so we expect that, if CURBD can correctly determine the functional connectivity levels between all pairs of regions, it should assign the highest levels to the pair R1-R2. Moreover, we applied a current injection to area R1 (Fig 2B) in order to test if CURBD is also able to detect not only information transfer - and therefore quantify functional connectivity - in baseline conditions, but also capture enhanced information transfer following a current injection mainly affecting strongly interconnected areas (in this case R1 and R2). We chose to apply current injection in R1 in order to contrast functional and anatomical connectivity, by assessing the elicited responses in R2. If we had opted for applying current in R2, the responses elicited in R1 might in fact have solely been due to the strong anatomical connectivity from R2 to R1. As expected, a current injection in R1 increased the overall firing rate in all regions (since all regions are interconnected), but significantly more strongly in R2, in line with the stronger interconnection between R1 and R2. (Fig. 2B). When fitted to simulated data taken from 20 neurons per area, CURBD learned to reproduce the simulated data, for what pertains to both single unit activity (Fig 2C-left) and population-level activity patterns (Fig 2C-right).

We then proceeded to test the reliability of CURBD in estimating inter-areal connectivity, when considering a minimal number of neurons (one per area, for a total of 4 neurons). Here, we chose one neuron per area and applied the CURBD algorithm 1000 times (with a different random seed in each iteration), in order to determine if the connectivity estimates were consistent across different runs of the algorithm. We first applied this approach to the baseline activity (500 ms of spontaneous activity, Fig 3A), obtaining - as expected - significantly higher levels of connectivity estimates between R1 and R2 compared to all other pairs of regions (p<0.001, one way ANOVA with post-hoc Tukey correction). Therefore, CURBD applied to spontaneous network activity reflected the strength of anatomical connectivity between areas.

Moreover, we also tested if this result was specifically dependent on the 4 neurons we chose to apply the algorithm in the previous test, as well as on the number of neurons included in the computations. Specifically, we varied the number of neurons provided to CURBD per region (e.g 1 neuron per area, 2 neurons per area). For each selection of the number of neurons we ran the CURBD algorithm 500 times, each time randomly selecting a set of neurons from each of the simulated regions. The estimated level of connectivity remained rather constant independently of the number of neurons (Fig 3B), showing that, even when including very few neurons, the strength of functional connectivity between areas can be effectively estimated via CURBD (see also [(Perich et al., 2020)](https://www.zotero.org/google-docs/?fTKKvI)).

Then, we applied the same approach to simulated neural activity after current injection in R1. This enabled us to test to what extent CURBD captures not only anatomical connectivity (that will vary across brain states except for some minor effects due to ongoing synaptic plasticity), but also effective connectivity, i.e. information transfer between regions. Indeed, during a 500 ms period of current-induced enhanced activity, CURBD estimated a level of functional connectivity that is generally higher in magnitude (Fig 3C) in comparison to baseline, and in particular between R1 and R2, in line with the observed changes in spiking activity. Specifically, connectivity between R1 and R2 was significantly stronger than that between all other pairs of regions (p<0.001, one way ANOVA with post-hoc Tukey correction). Moreover, when varying the number of provided units per region, analogously to what we observed for spontaneous activity, there were no strong variations on the estimated levels of connectivity (Fig 3D).

Overall, these results indicate that CURBD can reliably capture information transfer between brain regions even when few neurons can be recorded. Of relevance, the inclusion criterion we set for the in vivo recordings (see the Sample Size section) far exceeds the minimum number of one neuron per region observed in simulations to take into account physiological variability in neural activity (see also the results of the pilot analysis presented in Fig 5). Besides validating CURBD, this analysis clarifies that this method estimates effective connectivity between areas, based on how activity in one brain region determines patterns of spiking activity in another region. This is of course influenced by the underlying anatomical connectivity, but is also dependent on the actual engagement of individual brain regions in information processing and communication. However, as the term effective connectivity is often implied to include the estimation of causal effects - that we cannot estimate in the absence of an interventional approach using e.g. optogenetics - we will instead stick to the term functional connectivity in the rest of the manuscript.

**Statistical plan**

**Hypothesis testing**

For all the defined hypotheses, the dependent variable is, as discussed above, the estimated functional connectivity between pairs of neurons located in different areas (see Estimation of connectivity for more details). Specifically, for each 5 s epoch, we will compute the current estimation over the whole epoch for all pairs of neurons located in different areas (Fig 4A). This will give us an estimate of the inter-areal connectivity between all the recorded areas in a given 5 s epoch (Fig 4B). This will represent the sample unit for all the statistical analyses described in the following sections. We will test the following hypothesis regarding the functional connectivity among brain regions in NREM and REM sleep, respectively, independently:

**Hypothesis 1 (H1):**

Hypothesis 1 (H1): A significant fraction of NREM epochs displays a structure of interareal FC that is indistinguishable from what is observed on average in REM and wakefulness. Null hypothesis for NREM (NREM_H0): less than 5% of NREM epochs exhibit level of FC comparable to that observed in REM and/or wakefulness; for those NREM epochs with average FC values comparable to those observed in REM and wakefulness, a structure that is different from that observed in REM and/or wakefulness is observed. Of relevance, we will separately compare NREM to wakefulness and REM, but have no a priori hypothesis about any possible differences between wakefulness and REM.

In view of previous studies [(Olcese et al., 2016, 2018)](https://www.zotero.org/google-docs/?rDZnbw), we expect that FC will not significantly vary for pairs of neurons located in the same brain region. Therefore, we will here focus on pairs of neurons located in different regions. We will analyze the data obtained in different experimental conditions (Habituation, Fear conditioning and Fear test). We will test H1 at the level of individual recording sessions and we expect the effect to be present independently from experimental conditions taking place in the awake state. To test H1, we will first average all FC values obtained in a given epoch for all inter-areal pairs of neurons. This will allow us to compute a global value of inter-areal FC at single-epoch resolution. Based on previous studies [(Massimini et al., 2005; Olcese et al., 2016, 2018)](https://www.zotero.org/google-docs/?esfkUb), we expect that the global value of inter-areal FC will be, on average, lower during NREM than in either REM and wakefulness. State-dependent functional connectivity between cortical areas is expected to drop during NREM sleep, in comparison to wakefulness, both in view of empirical evidence [(Massimini et al., 2005; Storm et al., 2017)](https://www.zotero.org/google-docs/?MR4EwH) as well as theoretical considerations [(Koch et al., 2016; Tononi et al., 2024)](https://www.zotero.org/google-docs/?3n73If). In contrast, there is evidence that the subcortical-cortical communication (e.g from the hippocampus to cortical areas), can be preserved or even enhanced during NREM sleep [(Chen & Wilson, 2023; Ji & Wilson, 2007; Klinzing et al., 2019; Louie & Wilson, 2001)](https://www.zotero.org/google-docs/?q4EDBC). These specific state-dependent functional connectivity changes across cortical and subcortical regions have, however, been shown to be dependent on the connectivity measure and time scale under consideration [(Olcese et al., 2016, 2018)](https://www.zotero.org/google-docs/?f5mvhg). In view of this tight relationship between the specific details of the method that is used and the results that are obtained, we will here focus on quantifying how CURBD-estimated connectivity varies across brain states, in terms of average values and structure, without considering whether distinct effects might occur based on which specific pairs of areas are considered. Overall, based on previous studies [(Massimini et al., 2005; Olcese et al., 2016)](https://www.zotero.org/google-docs/?z1tQet), we expect a global drop of connection strength during NREM sleep in comparison to REM and wakefulness. Nevertheless, we cannot exclude that connection strength between some areas (e.g. between cortical areas and hippocampus) might follow different trends. This, however, would not be a problem for what pertains testing our hypotheses, since we will investigate if the FC structure varies within brain states, and not specifically how it varies.

We will test if FC strength varies across brain states using a repeated measurement ANOVA with post-hoc Tukey correction (alpha: 0.05), or a corresponding non-parametric test in case the assumptions of normality are not satisfied. This is a verification that the FC values we will obtain conform to the expected global changes across brain states, and is a prerequisite for testing H1. In case this analysis does not confirm the expected results, we will not pursue H1 and only present the results for average FC values.

The next step will be to determine, within individual recording sessions, the fraction of NREM epochs with a global level of inter-areal FC high enough to be comparable with the values observed in REM and wakefulness (H1, Fig 4C). To this aim, we will first compute the fraction of NREM epochs with an average inter-areal FC (aiFC) in the top 25th percentile for NREM and in any case higher than the 5th percentile of the distribution of aiFC values in REM (wakefulness). In view of the pilot results we have obtained (see the “Pilot Results”) section, this will allow to select NREM epochs whose aiFC values, irrespective of the source of the variability in FC values, are likely to be in the range observed during REM (or wakefulness) rather than NREM. If, nevertheless, the fraction of NREM epochs with aiFC values higher than the 5th percentile of the distribution of aiFC values in REM (wakefulness) is lower than 5% in more than 50% of the recording sessions, we will conclude that NREM_H0 cannot be rejected. Otherwise, we will test, in the recording sessions showing a significant overlap, if the NREM epochs with a high aiFC simply reflect a NREM network architecture with stronger FC values, or rather include epochs with a REM-like (wakefulness-like) network architecture (H1).

To test H1, we will train a classifier (support vector machine) to discriminate, on the basis of the values of individual directed connectivity values between inter-areal pairs of neurons, whether NREM epochs in the high tail of the aiFC distribution - high-aiFC, defined as described in the previous paragraph - are classified as either NREM or REM (wakefulness). The classifier will be trained on high-aiFC NREM epochs and on all REM (wakefulness) epochs using a modified leave-one-out cross-validation scheme in which only the NREM epochs in the high tail of the aiFC distribution are individually tested. This procedure will deliver a ground truth accuracy value for the classification of NREM vs. REM (wakefulness) epochs with similar values of aiFC. To test if this accuracy value reflects H1 or NREM_H0, we will repeat the cross-validation procedure by shuffling the labels of NREM and REM (wakefulness) epochs with comparable values (i.e., separately within 1 out of 10 equipopulated bins in which the range of REM (wakefulness) aiFC values will be subdivided). More precisely, assuming there are N NREM epochs with aiFC values in the high tail of the distribution, we will repeat the leave-one-out scheme by identifying N REM (wakefulness) epochs with values within the range of aiFC values observed in the selected NREM epochs. At each iteration of the cross-validation, 1 NREM epoch will be left out for testing. For the other N-1 epochs, sleep stage labels will be randomly shuffled with those of the selected REM (wakefulness) epochs with corresponding aiFC range (separately for each of 10 equipopulated bins in which the range of REM (wakefulness) aiFC value will be subdivided, i.e. each bin will have a width of ten percentiles of the distribution of aiFC values). The shuffling procedure will be repeated 1000 times. This shuffling procedure will allow us to obtain, for each recording session, 1000 estimates of classification performance for NREM and REM (wakefulness) epochs not only with comparable aiFC values, but also indistinguishable network architecture. In case the ground truth accuracy value falls within the 95% confidence interval of the shuffled estimates, this will mean that there is, within NREM epochs, a significant fraction of epochs whose inter-areal network architecture is indistinguishable from that observed in REM (wakefulness) - Fig 4C - therefore supporting H1. A p-value will be calculated as the fraction of instances in which a classifier trained on the shuffled dataset shows an accuracy greater than ground-truth classification accuracy.

The procedure described above will be repeated three times, respectively on NREM epochs with FC values in the top 25%, 15% and 5% of observed NREM values. This will allow us to test H1 as a function of the strength of aiFC observed in NREM. If ground truth accuracy value falls within the 95% confidence interval of the shuffled estimates for a given range of high-aiFC values ((top 25%, 15% and 5%), this will be seen as a confirmation of H1 and rejection of NREM_H0. If the ground truth accuracy is instead higher than the 95% confidence interval of the shuffled estimates - to be separately estimated for each of the three ranges of high-aiFC (top 25%, 15% and 5%) - we will not be able to reject NREM_H0.

To rule out the possibility that failure to reject H1 might stem not from a confirmation of our hypothesis, but from other aspects, such as an insufficient sample size, we will train a classifier to discriminate NREM epochs in the 5th percentile for FC strength from REM (or wakefulness) epochs in the 95th percentile for FC strength. As these epochs are those assumed to differ the most in terms of FC strength, we expect that a classifier will not be able to correctly (i.e. above chance) discriminate to which brain state each epoch belongs only if sample size is too low or if other aspects (e.g. noise in CURBD estimates) are insufficient to obtain sufficiently high accuracy. Ground-truth classification accuracy will be compared to accuracy of a classifier trained after randomly shuffling the labels of NREM and REM (or wakefulness) epochs. If no difference is observed between the two (i.e. if ground-truth accuracy will fall within the 95% confidence interval of accuracy for classifiers trained on the shuffled datasets) we will exclude the session.

Finally, we will combine the results - separately for each of the three high-aiFC ranges under consideration - across multiple recording sessions. In case the sample size analysis shows that we have enough power to draw meaningful conclusions from different types of sessions (e.g fear conditioning vs. habituation), we will perform this analysis. Otherwise, we will only compute a combined p-value for all sessions (via Fisher method). If the combined p-value across all sessions is larger than 0.05, we will interpret this as a confirmation of H1 and rejection of NREM_H0 (see Fig. 4B). Conversely, a combined p-value lower than 0.05 will not allow us to reject NREM_H0. In order to control for the possibility of individual animals strongly influencing the outcome of our analysis, we will also apply Fisher’s method to separately combine p-values within each animal. We will report the combined p-values per animal and verify that comparable results can be obtained across all animals. In view of the limited number of animals, in case we find that results differ between animals, we will conclude that our dataset is underpowered.

**Hypothesis 2 (H2):**

Hypothesis 2 (H2): A significant fraction of REM epochs displays a structure of interareal FC that is comparable to what is observed on average in NREM. Null hypothesis for REM (REM_H0): <5% of REM epochs generally exhibit level of FC comparable to that observed in NREM; for those REM epochs with average FC values comparable to those observed in NREM, a structure that is different from that observed in NREM is observed. To test this hypothesis (analogous to H1, but for REM sleep), we will follow the exact same procedures as for H1. We will train a classifier (support vector machine) to discriminate, on the basis of the values of individual directed connectivity values between interareal pairs of neurons, whether REM epochs in the low tail of the aiFC distribution - those epochs whose aiFC value is in the bottom 25th, 15th or 5th percentile of aiFC values for REM epochs (each of these three ranges will be separately analyses, similarly to H1) and in any case lower than the 95th percentile of aiFC values for NREM epochs - are classified as either NREM or REM.

###

### **Sampling plan**

For this study, we will be utilizing an existing dataset (see Table 5), in light of the ethical principle of reduction in animal research. This significantly lessens the number of animals used by avoiding unnecessary replication of data collection. Moreover, leveraging existing data promotes efficient use of resources and prevents potential biases introduced during data collection. In case our sample size analysis concludes that the proposed dataset does not have enough statistical power to draw the conclusions we will add different recording sessions that were obtained from the same experiment. Here we propose carrying the investigation on the first three days from a fear conditioning and extinction paradigm, but two additional recording sessions (extinction training and extinction test) are available in case it is needed. These sessions have not been pre-processed yet but may yield additional data, pending quality control.

##

## Our dataset was collected through multi-area tetrode recordings [(Olcese et al., 2016, 2018)](https://www.zotero.org/google-docs/?FeAbdK). This is a technique that enables the simultaneous recording of a more limited number of neurons, when compared to recently introduced techniques (e.g. chronic Neuropixels probe recordings [(van Daal et al., 2021)](https://www.zotero.org/google-docs/?ZXg6na)). Furthermore, we are only able to determine in which brain regions recorded neurons were located, but not in which area subdivision (e.g. cortical layer), nor to which neuronal subpopulation a neuron belongs (e.g. which type of interneuron).

##

## In view of the relatively low number of recorded neurons, we have decided not to subdivide them in subpopulations - e.g. between putative excitatory or inhibitory neurons based on the action potential waveforms [(Olcese et al., 2013; Oude Lohuis et al., 2022)](https://www.zotero.org/google-docs/?5KvsuV). Since our analysis is based on comparing patterns of inter-areal FC (i.e., averaged across pairs of neurons), we will include both putative single units and high-quality MUA (see also the “Spike Detection” section). Furthermore, as explained earlier, we will not aim to quantify or interpret connectivity patterns between individual neurons. Instead, we will only quantify the average connection strength measured for neurons located between brain regions. This approach, that we successfully employed in previous studies [(Olcese et al., 2016, 2018)](https://www.zotero.org/google-docs/?M4YYYc), will thus prevent the risk of drawing unwarranted conclusions from a limited number of recorded neurons. Therefore, this study will only allow us to draw conclusions about how brain states modulate pairwise functional connectivity between areas. A microcircuit-level characterization of such modulation may be performed at a later stage, based on the results that we will obtain. Furthermore, all the four areas from which recordings were done will be included. In fact even if not all the recorded areas are expected to contribute to establishing the level consciousness, previous studies showed that inter-areal connectivity is modified across most areas [(Olcese et al., 2016, 2018)](https://www.zotero.org/google-docs/?OYEbQC). Thus, independently of whether connectivity between areas is depressed (as is expected between cortical areas during NREM sleep compared to wakefulness) or enhanced (as may occur between hippocampus and cortex during NREM compared to wakefulness), we will nonetheless be able to use the recorded data to address our key hypothesis about the heterogeneous nature of brain states. Crucially, as we will discuss in the section “Sample size”, the number of neurons recorded in each session is estimated to be sufficient to obtain valid CURBD estimates. Importantly, in view of the fact that different set of neurons were recorded during each recording sessions (as tetrodes were independently advanced through brain tissue by at least 250 μm), but also following the typical convention in the field [(Bos et al., 2019; Dorman et al., 2023; Olcese et al., 2016, 2018)](https://www.zotero.org/google-docs/?Yi34Vt) and standard neuroscientific practice, each recording session should in fact be considered a separate observation [(Asaad & Sheth, 2024)](https://www.zotero.org/google-docs/?0fx3an). Thus, even if data was collected from only three animals, we have in fact access to at least seven separate recording sessions, that we may extend based on the quality of extra recording sessions collected during fear extinction sessions if necessary (see also earlier sections).

## **Exclusion Criteria**

We applied two key exclusion criteria in our analysis. First, following our pilot analysis on the effectiveness of CURBD in fitting the collected data as a function of the number of neurons (see the Sample Size section), we will exclude every recording session where the total number of neurons is less than 10. Second, we will exclude epochs in which less than two neurons were firing, due to their potential to generate noisy and statistically insufficient data. Third, we excluded epochs where sound stimulation occurred, along with the immediately adjacent epochs, to minimize potential confounding effects and to focus exclusively on spontaneous activity changes during sleep. Fourth, in cases in which there are no significant values for interareal connectivity across all considered brain states, we will interpret this as an indication of no observable communication between the considered areas. We will exclude the corresponding session from further analysis.

**Sample size**

In view of the nature of the analyses we will perform, we are not able to make an estimation of the effect size we will obtain. For this reason, we could not perform a classical power analysis, but instead focused on determining if we have a sufficient sample size (number of neurons, epochs, sessions) to draw significant conclusions. We have separately addressed this issue for what pertains a) the minimum number of neurons required by CURBD to obtain reliable estimates of FC, b) the number of epochs required to reliably estimate different FC structure between brain states and c) the number of recording sessions needed overall to obtain reliable results.

Number of neurons in each recording session

Even if CURBD does not theoretically require a minimum amount of neurons to provide meaningful current estimations [(Perich et al., 2020)](https://www.zotero.org/google-docs/?47YlVE), we nonetheless performed a pilot analysis meant to determine which recording sessions contain a number of neurons high enough to obtain a reliable CURBD-based estimate of inter-areal functional connectivity.

In order to avoid performing any analysis on the portion of the dataset that we aim to use in the following phase of the project, we decided to focus on epochs that we planned to exclude from the main analysis. In detail, our dataset included recordings performed in rats that were also exposed to auditory stimuli during sleep. In the main analyses we will focus on spontaneous spiking activity, and remove all epochs that contain sound exposure. For this additional analysis we instead extracted all the epochs during one single example REM sleep session in which a specific sound (CS+) was played (Fear test, table 5). In other words, we only used epochs that will be excluded from the main analysis, from one recording session only. Then, we subsampled data from the selected recording session by randomly selecting a number of neurons ranging from 4 to 24. For each value of the number of neurons, randomly sampled 500 different combinations of neurons, and for each of these combinations we obtained CURBD estimates. Then, we quantified the partial variance (pVar) that each fitted model was able to explain about the recorded data. This analysis revealed that, when at least 10 neurons were included in a subsampled dataset, any additional neuron did not improve the variance explained by the model (see Fig. 5). Therefore, we excluded sessions that did not have a minimum of 10 units, as they would lead to suboptimal CURBD estimates.

Number of epochs in each recording session

For what instead pertains to the number of sleep epochs in each session, the availability of enough data to potentially reject the null hypothesis is ensured by the shuffling procedure we discussed earlier. Specifically, our assessment is focused on the availability of a sufficiently large dataset to train the classifier used in testing H1/2. To test this, we will harness the classifier’s ability to discriminate between two brain states by using a similar leave-one-out scheme as that described in the “Hypothesis testing” section. The classifier will be trained on all the available epochs for two states (e.g. NREM and REM) and the performance will be tested via a cross-validated leave-one-out scheme using all epochs (and not just the epochs in the tail of the aiFC distribution. The performance of the classifier will be tested against that obtained after shuffling the labels of behavioral state (same procedure as described in the “Hypothesis testing section”. If the classifier is able to discriminate which epoch belongs to which state with an accuracy significantly higher than chance (i.e. larger than the 95th percentile of accuracy values obtained on the shuffled training sets) we will conclude that the number of epochs is sufficient to apply the proposed approach. This verification will be done for all recordings and between all pairs of brain states. Any session and pair of brain states with a classification accuracy not higher than chance will be excluded from further analyses. The results of this analysis will be reflected in the stage 2 manuscript by adding a column to table 5 with the indication, for each recording session, of whether a sufficient number of epochs to compare different brain states is present or not.

Thus, by comparing the ground truth classification accuracy to the value obtained with the shuffling procedure we will test if we can reject or not H0. In case H0 can be rejected, this will necessarily mean that we have enough epochs to compute valid classification estimates and that H1 (or H2) are verified. Otherwise, we will not be able to make any claim about H1 or H2.

Number of recording sessions

Finally, we will develop a simulation-based approach to estimate the power of the meta-analysis performed when combining p-values obtained from individual recording sessions (Fig. 4B). This is necessary because, in view of the novel nature of our research question and methodology, we are unable to make any a priori assumption of the effect size and variability of the effect we will see. Therefore, to avoid any risk of circularity [(Hoenig & Heisey, 2001)](https://www.zotero.org/google-docs/?AIsvXx), a simulation-based power analysis will be performed based on a set of pilot analyses run on 3 recording sessions [(Fridley et al., 2010)](https://www.zotero.org/google-docs/?nHUOgq). First, for each of these sessions, we will identify the classification accuracy corresponding to a p-value of 0.05. Then, we will randomly resample data (with replacement) from these 3 sessions to generate surrogate datasets consisting of 7 sessions (same number of sessions as in our dataset). For each session in a surrogate dataset, we will compute the p-value associated to the accuracy level corresponding, in the original recording session from which the data was drawn, to a p-value of 0.05. For each surrogate dataset a combined p-value will be computed via Fisher’s method. The procedure will be performed 1000 times to compute a statistical power. If power results to be higher than 80% we will continue with the analysis, otherwise we will conclude that we do not have sufficient data to draw any conclusion, i.e. that our dataset is underpowered. In case the dataset is deemed as underpowered, we can expand it with other remaining sessions of our behavioral paradigm (see also the Sampling plan). Conversely, this analysis might show that we have enough data to quantify possible differences between the various types of sessions (e.g. fear conditioning independently from baseline controls). In this case we will analyze the different types of sessions separately.

**Limitations**

The proposed study aims to re-use an existing, previously collected dataset. This prevents the need to perform novel experiments and is thus in line with the well-recognized need to reduce the number of animal experiments. Nevertheless, this approach also has some downsides. First of all, we will not be able to collect novel data. Therefore, a risk is that the available sample size might result to be insufficient to draw statistically significant conclusions. In particular, we are aware that the data was collected from only three animals, each undergoing a set of experimental conditions unrelated to the scientific objective of the current study. This sample size is comparable to what is often done for studies involving multi-area tetrode recordings (see also the “Sampling plan” section for more details). However, we cannot exclude that distinct animals or experimental conditions might show different results. Furthermore, we are also aware that our recordings yielded a low number of neurons, especially when compared to novel technologies such as Neuropixels probes. However, these methods were not yet available when the experiment we will analyze here was performed. Based on the modeling study we performed, we are confident that the CURBD method will yield reliable results even with a limited number of neurons. However, we expect that our study will be followed up by new investigations involving large-scale recordings, that will be able to further characterize how FC is modulated by brain states.

**Pilot Analyses**

In order to perform a preliminary test of the analytical procedures described here, we have performed a pilot analysis on single units recorded from one randomly selected recording session (animal r20, fear test session). First, we aimed to test if the assumptions underlying the ability to perform H1 (i.e. of higher inter-areal FC values - in absolute value - in wakefulness compared to NREM sleep) were verified. This was verified (Fig 6A; p<0.05, Mann-Whitney U test), although we also observed a considerable overlap between FC values observed in wakefulness and NREM. Such a high overlap in FC values could reflect a limited change in FC values between wakefulness and NREM, but also be indicative of a low reliability of CURBD-estimated FC. To better understand this, we first computed inter-areal FC strength while excluding connections to and from the hippocampus - since cortico-hippocampal communication is known to be preserved in NREM sleep compared to wakefulness [(Olcese et al., 2016, 2018)](https://www.zotero.org/google-docs/?FB6xpP); we observed that the overlap between FC values - quantified by looking at the fraction of NREM FC values falling within each quartile of the distribution of wake FC values - was lower compared to when all the recorded areas were included (Fig 6B). We then focused on epochs in which the highest difference in FC between wakefulness and NREM could be expected. Specifically, we focused on the 10% of NREM epochs with the highest slow wave activity, obtained by calculating the LFP power between 0.5 and 4Hz, and the 10% of wakefulness epochs with the highest motor activity, obtained by calculating the area under the curve of accelerometer activity in each epoch. As expected, the distributions of FC values were more different (both in terms of average value and overlap between the distributions) compared to when we included all epochs in the analysis (Fig 6C), an effect that was even more prominent when we excluded connections to and from the hippocampus - Fig 6D. Conversely, and also in line with previous studies [(Olcese et al., 2016, 2018)](https://www.zotero.org/google-docs/?4mkakL), no significant difference was observed between wakefulness and sleep for intra-real FC strength (Fig 6E). Finally, and also in line with our assumption, a significant difference was observed for inter-areal FC coupling between NREM and REM (with stronger FC during REM - Fig 6F; p<0.05, Mann-Whitney U test).

Finally, in order to test the full analytical procedure to compare FC structure between brain states, applied the analytical procedure, as described in the “Hypothesis Testing” section to compare the FC structure between high-aiFC NREM epochs and wakefulness epochs, separately for the NREM epochs with FC values in the top 25% and top 5% (Fig 6G and 6H, respectively). In both cases, the ground truth accuracy is higher than the 95% confidence interval of accuracy values for classifiers trained on the shuffled dataset. This pilot analysis is important to gauge the range of accuracy values obtained for both ground truth and shuffled datasets (note the unbalanced nature of the dataset, with more wakefulness epochs compared to high-aiFC epochs). Although this pilot analysis reject H1 for this specific recording session (cf. Fig 4C), we refrain from drawing any conclusion.

# **Data availability**

# In adherence to open science principles and in agreement with the journal's policies, we commit to make our raw data and research materials available upon acceptance of our Stage 2 manuscript.

# **Code availability**

In compliance with the requirements for Registered Reports and in accordance with the principles of open science, we are committed to publicly sharing all code associated with this research upon acceptance of our Stage 2 manuscript. The code includes scripts used for data simulation, power analyses, and pilot data analysis.

The code will be accessible via our dedicated repository on GitHub, and this link will be provided in the final manuscript. We understand that the code needs to be made available for peer-review purposes and agree to an embargo on public access until Stage 2 acceptance. This ensures a rigorous review process while maintaining our commitment to full transparency and reproducibility in scientific research.

#

#

# **References**

[Asaad, W. F., & Sheth, S. A. (2024). What’s the n? On sample size vs. subject number for brain-behavior neurophysiology and neuromodulation. *Neuron*, *112*(13), 2086–2090. https://doi.org/10.1016/j.neuron.2024.04.033](https://www.zotero.org/google-docs/?xifdO5)

[Aserinsky, E., & Kleitman, N. (1953). Regularly Occurring Periods of Eye Motility, and Concomitant Phenomena, During Sleep’. *Science*, *118*.](https://www.zotero.org/google-docs/?xifdO5)

[Bagur, S., Lefort, J. M., Lacroix, M. M., de Lavilléon, G., Herry, C., Chouvaeff, M., Billand, C., Geoffroy, H., & Benchenane, K. (2021). Breathing-driven prefrontal oscillations regulate maintenance of conditioned-fear evoked freezing independently of initiation. *Nature Communications*, *12*(1), 1–15. https://doi.org/10.1038/s41467-021-22798-6](https://www.zotero.org/google-docs/?xifdO5)

[Billeh, Y. N., Cai, B., Gratiy, S. L., Dai, K., Iyer, R., Gouwens, N. W., Abbasi-Asl, R., Jia, X., Siegle, J. H., Olsen, S. R., Koch, C., Mihalas, S., & Arkhipov, A. (2020). Systematic Integration of Structural and Functional Data into Multi-scale Models of Mouse Primary Visual Cortex. *Neuron*, *106*(3), 388-403.e18. https://doi.org/10.1016/j.neuron.2020.01.040](https://www.zotero.org/google-docs/?xifdO5)

[Bos, J. J., Vinck, M., Marchesi, P., Keestra, A., Van Mourik-Donga, L. A., Jackson, J. C., Verschure, P. F. M. J., & Pennartz, C. M. A. (2019). Multiplexing of Information about Self and Others in Hippocampal Ensembles. *Cell Reports*, *29*(12), 3859-3871.e6. https://doi.org/10.1016/j.celrep.2019.11.057](https://www.zotero.org/google-docs/?xifdO5)

[Buccino, A. P., Hurwitz, C. L., Garcia, S., Magland, J., Siegle, J. H., Hurwitz, R., & Hennig, M. H. (2020). SpikeInterface, a unified framework for spike sorting. *eLife*, *9*, e61834. https://doi.org/10.7554/eLife.61834](https://www.zotero.org/google-docs/?xifdO5)

[Chen, Z. S., & Wilson, M. A. (2023). How our understanding of memory replay evolves. *Journal of Neurophysiology*, *129*(3), 552–580. https://doi.org/10.1152/jn.00454.2022](https://www.zotero.org/google-docs/?xifdO5)

[D’Ambrosio, S., Castelnovo, A., Guglielmi, O., Nobili, L., Sarasso, S., & Garbarino, S. (2019). Sleepiness as a Local Phenomenon. *Frontiers in Neuroscience*, *13*, 1086. https://doi.org/10.3389/fnins.2019.01086](https://www.zotero.org/google-docs/?xifdO5)

[Das, A., & Fiete, I. R. (2020). Systematic errors in connectivity inferred from activity in strongly recurrent networks. *Nature Neuroscience*, *23*(10), 1286–1296. https://doi.org/10.1038/s41593-020-0699-2](https://www.zotero.org/google-docs/?xifdO5)

[Dorman, R., Bos, J. J., Vinck, M. A., Marchesi, P., Fiorilli, J., Lorteije, J. A. M., Reiten, I., Bjaalie, J. G., Okun, M., & Pennartz, C. M. A. (2023). Spike-based coupling between single neurons and populations across rat sensory cortices, perirhinal cortex, and hippocampus. *Cerebral Cortex*, *33*(13), 8247–8264. https://doi.org/10.1093/cercor/bhad111](https://www.zotero.org/google-docs/?xifdO5)

[dos Santos Lima, G. Z., Lobao-Soares, B., Corso, G., Belchior, H., Lopes, S. R., de Lima Prado, T., Nascimento, G., França, A. C. de, Fontenele-Araújo, J., & Ivanov, P. C. (2019). Hippocampal and cortical communication around micro-arousals in slow-wave sleep. *Scientific Reports*, *9*(1), 1–13. https://doi.org/10.1038/s41598-019-42100-5](https://www.zotero.org/google-docs/?xifdO5)

[El-Baba, M., Lewis, D. J., Fang, Z., Owen, A. M., Fogel, S. M., & Morton, J. B. (2019). Functional connectivity dynamics slow with descent from wakefulness to sleep. *PLoS ONE*, *14*(12). https://doi.org/10.1371/journal.pone.0224669](https://www.zotero.org/google-docs/?xifdO5)

[Emrick, J. J., Gross, B. A., Riley, B. T., & Poe, G. R. (2016). Different Simultaneous Sleep States in the Hippocampus and Neocortex. *Sleep*, *39*(12), 2201–2209. https://doi.org/10.5665/sleep.6326](https://www.zotero.org/google-docs/?xifdO5)

[Finkelstein, A., Fontolan, L., Economo, M. N., Li, N., Romani, S., & Svoboda, K. (2021). Attractor dynamics gate cortical information flow during decision-making. *Nature Neuroscience*, *24*(6), 843–850. https://doi.org/10.1038/s41593-021-00840-6](https://www.zotero.org/google-docs/?xifdO5)

[Fridley, B. L., Jenkins, G. D., & Biernacka, J. M. (2010). Self-Contained Gene-Set Analysis of Expression Data: An Evaluation of Existing and Novel Methods. *PLoS ONE*, *5*(9), e12693. https://doi.org/10.1371/journal.pone.0012693](https://www.zotero.org/google-docs/?xifdO5)

[Hestrin, S., Sah, P., & Nicoll, R. A. (1990). Mechanisms generating the time course of dual component excitatory synaptic currents recorded in hippocampal slices. *Neuron*, *5*(3), 247–253. https://doi.org/10.1016/0896-6273(90)90162-9](https://www.zotero.org/google-docs/?xifdO5)

[Hoenig, J. M., & Heisey, D. M. (2001). The Abuse of Power: The Pervasive Fallacy of Power Calculations for Data Analysis. *The American Statistician*, *55*(1), 19–24.](https://www.zotero.org/google-docs/?xifdO5)

[Huber, R., Felice Ghilardi, M., Massimini, M., & Tononi, G. (2004). Local sleep and learning. *Nature*, *430*(6995), 78–81. https://doi.org/10.1038/nature02663](https://www.zotero.org/google-docs/?xifdO5)

[Huber, R., Ghilardi, M. F., Massimini, M., Ferrarelli, F., Riedner, B. A., Peterson, M. J., & Tononi, G. (2006). Arm immobilization causes cortical plastic changes and locally decreases sleep slow wave activity. *Nature Neuroscience*, *9*(9), 1169–1176. https://doi.org/10.1038/nn1758](https://www.zotero.org/google-docs/?xifdO5)

[Ji, D., & Wilson, M. A. (2007). Coordinated memory replay in the visual cortex and hippocampus during sleep. *Nature Neuroscience*, *10*(1), 100–107. https://doi.org/10.1038/nn1825](https://www.zotero.org/google-docs/?xifdO5)

[Jobst, B. M., Hindriks, R., Laufs, H., Tagliazucchi, E., Hahn, G., Ponce-Alvarez, A., Stevner, A. B. A., Kringelbach, M. L., & Deco, G. (2017). Increased Stability and Breakdown of Brain Effective Connectivity During Slow-Wave Sleep: Mechanistic Insights from Whole-Brain Computational Modelling. *Scientific Reports*, *7*(1), 4634–4634. https://doi.org/10.1038/s41598-017-04522-x](https://www.zotero.org/google-docs/?xifdO5)

[Joglekar, M. R., Mejias, J. F., Yang, G. R., & Wang, X.-J. (2018). Inter-areal Balanced Amplification Enhances Signal Propagation in a Large-Scale Circuit Model of the Primate Cortex. *Neuron*, *98*(1), 222-234.e8. https://doi.org/10.1016/j.neuron.2018.02.031](https://www.zotero.org/google-docs/?xifdO5)

[Juan, E., Arslan, C., Regnath, F., & Talamini, L. M. (2023). *Boosting sleep slow waves suppresses dreaming* [Preprint]. Neuroscience. https://doi.org/10.1101/2023.03.10.532054](https://www.zotero.org/google-docs/?xifdO5)

[Klinzing, J. G., Niethard, N., & Born, J. (2019). Mechanisms of systems memory consolidation during sleep. *Nature Neuroscience*. https://doi.org/10.1038/s41593-019-0467-3](https://www.zotero.org/google-docs/?xifdO5)

[Koch, C., Massimini, M., Boly, M., & Tononi, G. (2016). Neural correlates of consciousness: Progress and problems. *Nature Reviews Neuroscience*, *17*(5), 307–321. https://doi.org/10.1038/nrn.2016.22](https://www.zotero.org/google-docs/?xifdO5)

[Kuan, A. T., Bondanelli, G., Driscoll, L. N., Han, J., Kim, M., Hildebrand, D. G. C., Graham, B. J., Wilson, D. E., Thomas, L. A., Panzeri, S., Harvey, C. D., & Lee, W.-C. A. (2024). Synaptic wiring motifs in posterior parietal cortex support decision-making. *Nature*, *627*(8003), 367–373. https://doi.org/10.1038/s41586-024-07088-7](https://www.zotero.org/google-docs/?xifdO5)

[Lansink, C. S., Goltstein, P. M., Lankelma, J. V., McNaughton, B. L., & Pennartz, C. M. A. (2009). Hippocampus Leads Ventral Striatum in Replay of Place-Reward Information. *PLoS Biology*, *7*(8), e1000173. https://doi.org/10.1371/journal.pbio.1000173](https://www.zotero.org/google-docs/?xifdO5)

[LeDoux, J. E. (2014). Coming to terms with fear. *Proceedings of the National Academy of Sciences*, *111*(8), 2871–2878. https://doi.org/10.1073/pnas.1400335111](https://www.zotero.org/google-docs/?xifdO5)

[Louie, K., & Wilson, M. A. (2001). Temporally structured replay of awake hippocampal ensemble activity during rapid eye movement sleep. *Neuron*, *29*(1), 145–156. https://doi.org/10.1016/S0896-6273(01)00186-6](https://www.zotero.org/google-docs/?xifdO5)

[Lu, H., Zou, Q., Gu, H., Raichle, M. E., Stein, E. A., & Yang, Y. (2012). Rat brains also have a default mode network. *Proceedings of the National Academy of Sciences*, *109*(10), 3979–3984. https://doi.org/10.1073/pnas.1200506109](https://www.zotero.org/google-docs/?xifdO5)

[Marshall, L., Helgadóttir, H., Mölle, M., & Born, J. (2006). Boosting slow oscillations during sleep potentiates memory. *Nature*, *444*(7119), 610–613. https://doi.org/10.1038/nature05278](https://www.zotero.org/google-docs/?xifdO5)

[Massimini, M., Ferrarelli, F., Huber, R., Esser, S. K., Esser, S. K., Singh, H., Harpreet Singh, & Tononi, G. (2005). Breakdown of cortical effective connectivity during sleep. *Science*, *309*(5744), 2228–2232. https://doi.org/10.1126/science.1117256](https://www.zotero.org/google-docs/?xifdO5)

[Mejias, J. F., & Longtin, A. (2014). Differential effects of excitatory and inhibitory heterogeneity on the gain and asynchronous state of sparse cortical networks. *Frontiers in Computational Neuroscience*, *8*. https://doi.org/10.3389/fncom.2014.00107](https://www.zotero.org/google-docs/?xifdO5)

[Moreni, G., Pennartz, C. M. A., & Mejias, J. F. (2023). *Synaptic plasticity is required for oscillations in a V1 cortical column model with multiple interneuron types*. https://doi.org/10.1101/2023.08.27.555009](https://www.zotero.org/google-docs/?xifdO5)

[Moreni, G., Pennartz, C. M. A., & Mejias, J. F. (2024). *Cell type specific firing patterns in a V1 cortical column model depend on feedforward and feedback activity*. https://doi.org/10.1101/2024.04.02.587673](https://www.zotero.org/google-docs/?xifdO5)

[Nir, Y., Massimini, M., Boly, M., & Tononi, G. (2013). Sleep and Consciousness. In A. E. Cavanna, A. Nani, H. Blumenfeld, & S. Laureys (Eds.), *Neuroimaging of Consciousness* (pp. 133–182). Springer Berlin Heidelberg. https://doi.org/10.1007/978-3-642-37580-4_9](https://www.zotero.org/google-docs/?xifdO5)

[Nobili, L. (2012). Local aspects of sleep: Observations from intracerebral recordings in humans. *International Journal of Psychophysiology*, *85*(3), 356–357. https://doi.org/10.1016/j.ijpsycho.2012.06.177](https://www.zotero.org/google-docs/?xifdO5)

[Nunes, R. V., Reyes, M. B., Mejias, J. F., & De Camargo, R. Y. (2021). Directed functional and structural connectivity in a large-scale model for the mouse cortex. *Network Neuroscience*, *5*(4), 874–889. https://doi.org/10.1162/netn_a_00206](https://www.zotero.org/google-docs/?xifdO5)

[Oh, S. W., Harris, J. A., Ng, L., Winslow, B., Cain, N., Mihalas, S., Wang, Q., Lau, C., Kuan, L., Henry, A. M., Mortrud, M. T., Ouellette, B., Nguyen, T. N., Sorensen, S. A., Slaughterbeck, C. R., Wakeman, W., Li, Y., Feng, D., Ho, A., … Zeng, H. (2014). A mesoscale connectome of the mouse brain. *Nature*, *508*(7495), 207–214. https://doi.org/10.1038/nature13186](https://www.zotero.org/google-docs/?xifdO5)

[Olcese, U., Bos, J. J., Vinck, M., Lankelma, J. V., van Mourik-Donga, L. B., Schlumm, F., & Pennartz, C. M. A. (2016). Spike-Based Functional Connectivity in Cerebral Cortex and Hippocampus: Loss of Global Connectivity Is Coupled to Preservation of Local Connectivity During Non-REM Sleep. *Journal of Neuroscience*, *36*(29), 7676–7692. https://doi.org/10.1523/JNEUROSCI.4201-15.2016](https://www.zotero.org/google-docs/?xifdO5)

[Olcese, U., Bos, J. J., Vinck, M., & Pennartz, C. M. A. (2018). Functional determinants of enhanced and depressed interareal information flow in nonrapid eye movement sleep between neuronal ensembles in rat cortex and hippocampus. *Sleep*, *41*(11), 1–18. https://doi.org/10.1093/sleep/zsy167](https://www.zotero.org/google-docs/?xifdO5)

[Olcese, U., Esser, S. K., & Tononi, G. (2010). Sleep and Synaptic Renormalization: A Computational Study. *Journal of Neurophysiology*, *104*(6), 3476–3493. https://doi.org/10.1152/jn.00593.2010](https://www.zotero.org/google-docs/?xifdO5)

[Olcese, U., Iurilli, G., & Medini, P. (2013). Cellular and Synaptic Architecture of Multisensory Integration in the Mouse Neocortex. *Neuron*, *79*(3), 579–593. https://doi.org/10.1016/j.neuron.2013.06.010](https://www.zotero.org/google-docs/?xifdO5)

[Oude Lohuis, M. N., Pie, J. L., Marchesi, P., Montijn, J. S., de Kock, C. P. J., Pennartz, C. M. A., & Olcese, U. (2022). Multisensory task demands temporally extend the causal requirement for visual cortex in perception. *Nature Communications*, *13*(1), 2864. https://doi.org/10.1038/s41467-022-30600-4](https://www.zotero.org/google-docs/?xifdO5)

[Pandarinath, C., O’Shea, D. J., Collins, J., Jozefowicz, R., Stavisky, S. D., Kao, J. C., Trautmann, E. M., Kaufman, M. T., Ryu, S. I., Hochberg, L. R., Henderson, J. M., Shenoy, K. V., Abbott, L. F., & Sussillo, D. (2018). Inferring single-trial neural population dynamics using sequential auto-encoders. *Nature Methods*, *15*(10), 805–815. https://doi.org/10.1038/s41592-018-0109-9](https://www.zotero.org/google-docs/?xifdO5)

[Percie du Sert, N., Hurst, V., Ahluwalia, A., Alam, S., Avey, M. T., Baker, M., Browne, W. J., Clark, A., Cuthill, I. C., Dirnagl, U., Emerson, M., Garner, P., Holgate, S. T., Howells, D. W., Karp, N. A., Lazic, S. E., Lidster, K., MacCallum, C. J., Macleod, M., … Würbel, H. (2020). The ARRIVE guidelines 2.0: Updated guidelines for reporting animal research. *PLOS Biology*, *18*(7), e3000410. https://doi.org/10.1371/journal.pbio.3000410](https://www.zotero.org/google-docs/?xifdO5)

[Perich, M. G., Arlt, C., Soares, S., Young, M. E., Mosher, C. P., Minxha, J., Carter, E., Rutishauser, U., Rudebeck, P. H., Harvey, C. D., & Rajan, K. (2020). *Inferring brain-wide interactions using data-constrained recurrent neural network models* [Preprint]. Neuroscience. https://doi.org/10.1101/2020.12.18.423348](https://www.zotero.org/google-docs/?xifdO5)

[Potjans, T. C., & Diesmann, M. (2014). The Cell-Type Specific Cortical Microcircuit: Relating Structure and Activity in a Full-Scale Spiking Network Model. *Cerebral Cortex*, *24*(3), 785–806. https://doi.org/10.1093/cercor/bhs358](https://www.zotero.org/google-docs/?xifdO5)

[Rasch, B., & Born, J. (2013). About sleep’s role in memory. *Physiological Reviews*, *93*(2), 681–766. https://doi.org/10.1152/physrev.00032.2012](https://www.zotero.org/google-docs/?xifdO5)

[Salin, P. A., & Prince, D. A. (1996). Spontaneous GABAA receptor-mediated inhibitory currents in adult rat somatosensory cortex. *Journal of Neurophysiology*, *75*(4), 1573–1588. https://doi.org/10.1152/jn.1996.75.4.1573](https://www.zotero.org/google-docs/?xifdO5)

[Sanders, H., Ji, D., Sasaki, T., Leutgeb, J. K., Wilson, M. A., & Lisman, J. E. (2019). Temporal coding and rate remapping: Representation of nonspatial information in the hippocampus. *Hippocampus*, *29*(2), 111–127. https://doi.org/10.1002/hipo.23020](https://www.zotero.org/google-docs/?xifdO5)

[Siclari, F., Baird, B., Perogamvros, L., Bernardi, G., LaRocque, J. J., Riedner, B. A., Boly, M., Postle, B. R., & Tononi, G. (2017). The neural correlates of dreaming. *Nature Neuroscience*, *20*(6), 872–878. https://doi.org/10.1038/nn.4545](https://www.zotero.org/google-docs/?xifdO5)

[Spoormaker, V. I., Gleiser, P. M., & Czisch, M. (2012). Frontoparietal Connectivity and Hierarchical Structure of the Brain’s Functional Network during Sleep. *Frontiers in Neurology*, *3*, 80–80. https://doi.org/10.3389/fneur.2012.00080](https://www.zotero.org/google-docs/?xifdO5)

[Spruston, N., Jonas, P., & Sakmann, B. (1995). Dendritic glutamate receptor channels in rat hippocampal CA3 and CAl pyramidal neurons. *J. Physiol.* https://doi.org/10.1113/jphysiol.1995.sp020521](https://www.zotero.org/google-docs/?xifdO5)

[Storm, J. F., Boly, M., Casali, A. G., Massimini, M., Olcese, U., Pennartz, C. M. A., & Wilke, M. (2017). Consciousness Regained: Disentangling Mechanisms, Brain Systems, and Behavioral Responses. *The Journal of Neuroscience*, *37*(45), 10882–10893. https://doi.org/10.1523/JNEUROSCI.1838-17.2017](https://www.zotero.org/google-docs/?xifdO5)

[Storm, J. F., Klink, P. C., Aru, J., Senn, W., Goebel, R., Pigorini, A., Avanzini, P., Vanduffel, W., Roelfsema, P. R., Massimini, M., Larkum, M. E., & Pennartz, C. M. A. (2024). An integrative, multiscale view on neural theories of consciousness. *Neuron*, *112*(10), 1531–1552. https://doi.org/10.1016/j.neuron.2024.02.004](https://www.zotero.org/google-docs/?xifdO5)

[Tagliazucchi, E., Behrens, M., & Laufs, H. (2013). Sleep Neuroimaging and Models of Consciousness. *Frontiers in Psychology*, *4*. https://doi.org/10.3389/fpsyg.2013.00256](https://www.zotero.org/google-docs/?xifdO5)

[Tononi, G., Boly, M., & Cirelli, C. (2024). Consciousness and sleep. *Neuron*, *112*(10), 1568–1594. https://doi.org/10.1016/j.neuron.2024.04.011](https://www.zotero.org/google-docs/?xifdO5)

[Van Albada, S. J., Helias, M., & Diesmann, M. (2015). Scalability of Asynchronous Networks Is Limited by One-to-One Mapping between Effective Connectivity and Correlations. *PLOS Computational Biology*, *11*(9), e1004490. https://doi.org/10.1371/journal.pcbi.1004490](https://www.zotero.org/google-docs/?xifdO5)

[van Daal, R. J. J., Aydin, Ç., Michon, F., Aarts, A. A. A., Kraft, M., Kloosterman, F., & Haesler, S. (2021). Implantation of Neuropixels probes for chronic recording of neuronal activity in freely behaving mice and rats. *Nature Protocols*, *16*(7), 3322–3347. https://doi.org/10.1038/s41596-021-00539-9](https://www.zotero.org/google-docs/?xifdO5)

[Vyazovskiy, V. V., Olcese, U., Hanlon, E. C., Nir, Y., Cirelli, C., & Tononi, G. (2011). Local sleep in awake rats. *Nature*, *472*(7344), 443–447. https://doi.org/10.1038/nature10009](https://www.zotero.org/google-docs/?xifdO5)

[Wang, X.-J. (2002). Probabilistic Decision Making by Slow Reverberation in Cortical Circuits. *Neuron*, *36*(5), 955–968. https://doi.org/10.1016/S0896-6273(02)01092-9](https://www.zotero.org/google-docs/?xifdO5)

[Xiang, Z., Huguenard, J. R., & Prince, D. A. (1998). GABA_A_ receptor‐mediated currents in interneurons and pyramidal cells of rat visual cortex. *The Journal of Physiology*, *506*(3), 715–730. https://doi.org/10.1111/j.1469-7793.1998.715bv.x](https://www.zotero.org/google-docs/?xifdO5)


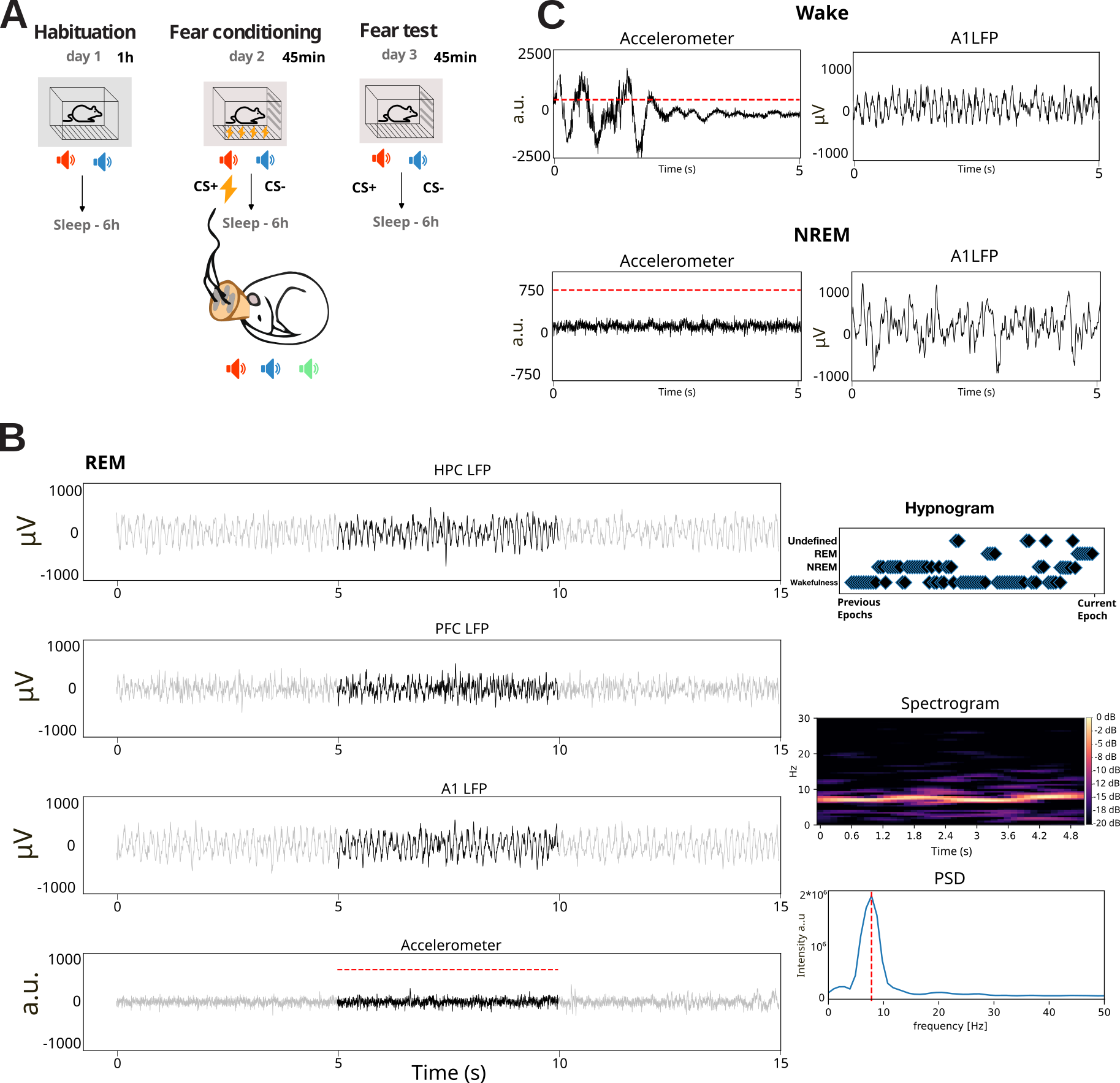


**Figure 1. Experimental procedure and scoring of behavioral states**. **A**. Day to day experimental procedure. Each experimental session (Habituation, Fear conditioning and Fear test) was followed by a session of sleep recordings. **B**. Exemplary traces of REM classification, indicating all the information that was used to perform sleep stage classification. In the left column (from top to bottom) LFP activity is depicted for the different channels (HPC, PFC, A1) that were used to monitor global ongoing activity. Gray traces indicate previous and subsequent sleep epoch, and the black trace is the epoch we are scoring. Below the LFP traces, accelerometer activity is depicted, with the dashed red line indicating the defined threshold for motor activity. On the right, from top to bottom: **Hypnogram** scoring of the previous 100 epochs; **spectrogram** showing the time frequency decomposition for the current epoch based on cortical (A1) activity (note that the oscillatory activity is centered on 8 Hz); **Power source density** (PSD) of the current epoch, indicating that most power is within the theta (6 - 12 Hz) band. **C**. Example LFP traces from one cortical channel (A1) recorded, respectively, during WAKE and NREM. Note the predominance of lower frequencies and higher amplitude during NREM, as well as the lower levels of head acceleration.


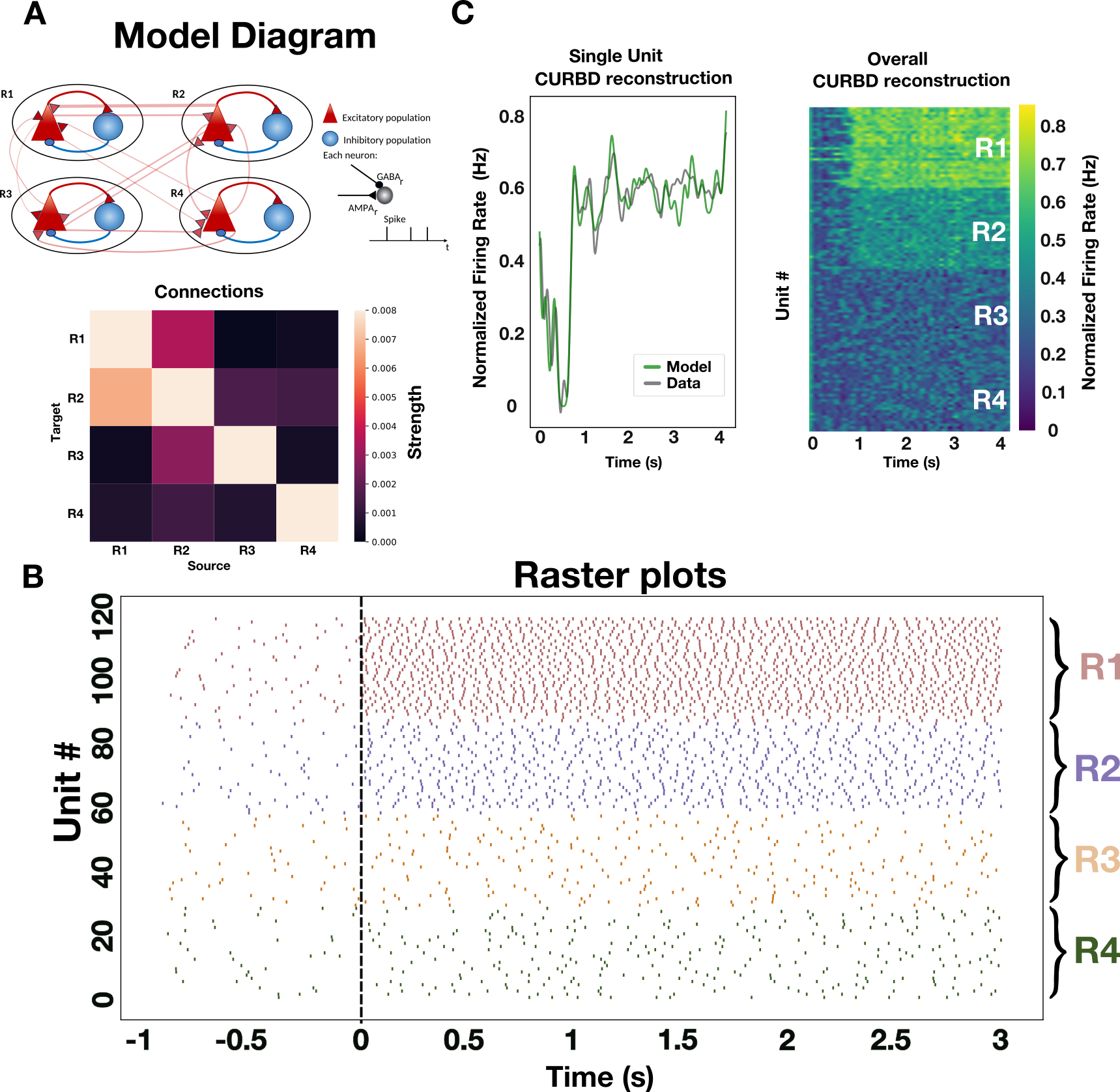


**Figure 2. Applying CURBD to a toy model. A.** Schematics of the model architecture. The model is composed of four areas (R1-R4). In each area one excitatory population (Red triangle) and one inhibitory population (blue circle) are present. In each area 85% of the population is of excitatory neurons and 15% of inhibitory neurons. In total, we simulated 5000 neurons. The thickness of the connections between the symbols represent the model connectivity strength. The heatmap shows the normalized connectivity strength (normalized number of synaptic connections) between each pair of regions. **B.** Raster Plot of spiking activity simulated for 4s. At time 1, a current of 30pA is applied to region 1 (R1). Activity in region 2 (R2), reflecting the stronger connectivity region R1, shows a higher firing rate as well following current injection, indicating the propagation of information between both areas.. **C**. Left. Following training, the CURBD reconstruction (green) of the activity of a single neuron reproduces the activity of the simulated neuron (gray). Activity is expressed as peristimulus time histogram (PSTH) traces. Right. CURBD reconstruction of the activity of all units provided to the algorithm (20 neurons/area). Note that, following current injection, the units in R1 and R2 exhibit higher firing rates, similar to what is observed in the simulated data.


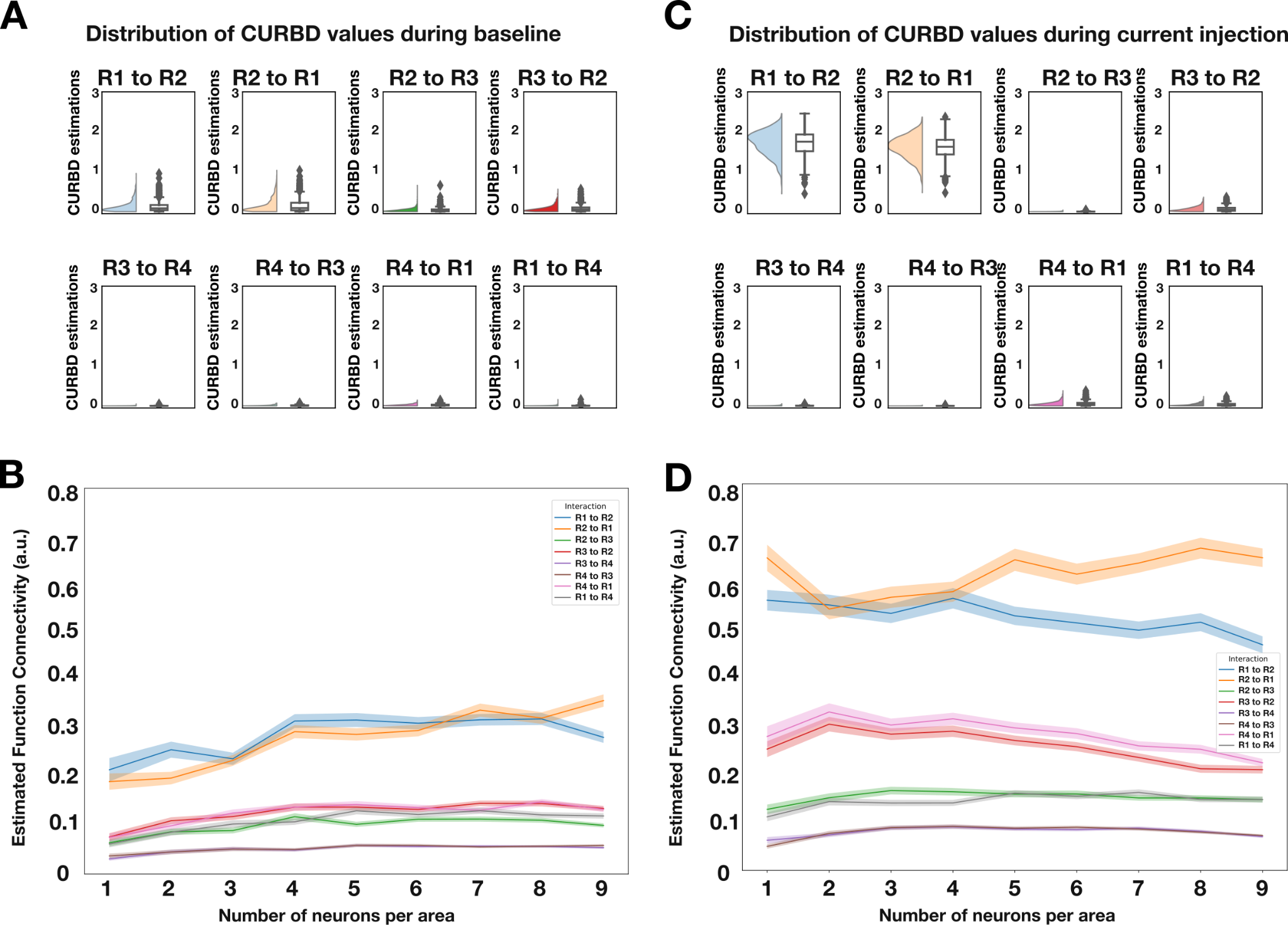


**Figure 3. CURBD-based estimates of connectivity reflect both anatomical as well as effective connectivity and are valid already with a very limited sample size. A.** For a selection of one unit per area (total of four units), we estimated the functional connectivity between the four different areas generated by the neural spiking model (Fig 2) across 1000 different runs of CURBD. For each iteration, we applied the CURBD algorithm to the same selection of units but using a different random seed, in order to observe if CURBD would be prone to spurious results when estimating connectivity with a low number of units. Estimated values during baseline (i.e spontaneous activity) are significantly higher between the pair R1-R2, matching the anatomical connectivity of the model. **B.** Varying the number of units per region leads to similar connectivity values. In order to assess if the results shown in panel A are dependent on the specific four units we chose for the initial simulation, we ran another set of tests. First, we varied the number of pooled cells from the model per region. Then, for each N selected units per region, we proceeded to randomly select N different cells on each run. We ran this test 500 times for each number of N units. Each line represents the average connectivity values observed after all the simulations, with SEM as the shaded area. **C**. Same as A, but for a period of activity after current injection. Note how the level of CURBD-estimated connectivity is significantly higher between R1 and R2 (and also higher compared to values observed during spontaneous activity), in line with the observed patterns of activity. **D.** Same as B, but for a period after current injection.


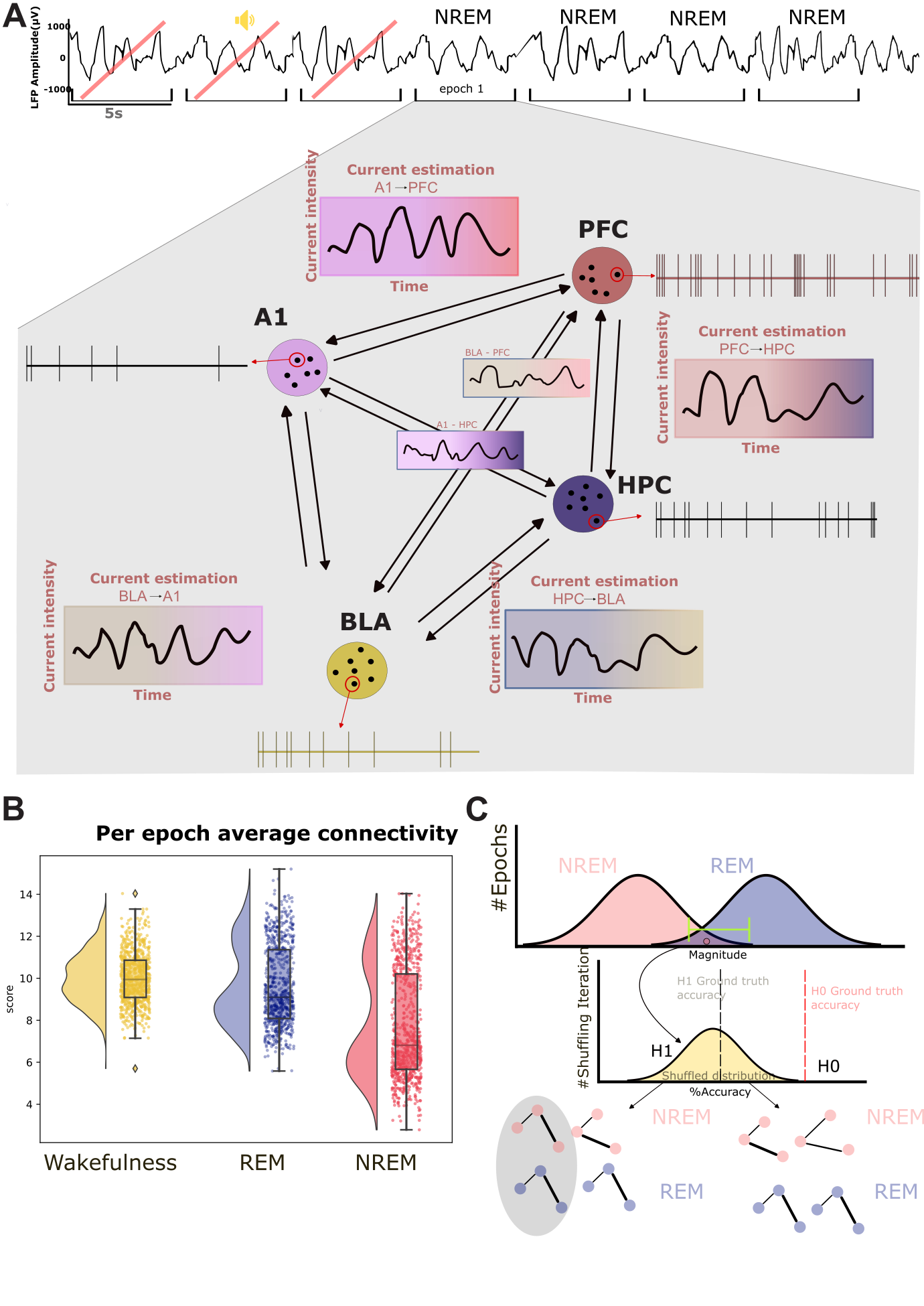


**Figure 4. Estimation of connectivity and hypothesis testing. A**. For each valid sleep epoch, single unit activity of all pairs of inter-region responsive neurons will be provided to the CURBD network. For each of these pairs we will calculate a 5s time series of current intensity, which is CURBDs’ measure of connectivity. **B.** By averaging, for each epoch, each of the current estimation time series, we will obtain a single score representing the measured connectivity on that epoch, between neurons of different regions.. **C.** We hypothesize that, despite global differences, a significant number of NREM epochs might contain connectivity values comparable to REM. For each epoch, through a leave-one-out approach performed on the higher tail of the NREM pairwise connectivity distribution, a classifier is trained to distinguish between NREM and REM epochs’ connectivity. The same procedure will be done by comparing NREM and wakefulness. By observing the distribution of the classifier performance, we can confirm H1 if the classifier returns a low ground truth accuracy. Similarly, if it returns a high accuracy, we can confirm H0. Importantly, this procedure will also take in account the shape of the network, instead of focusing solely on the average global values of all pairs.


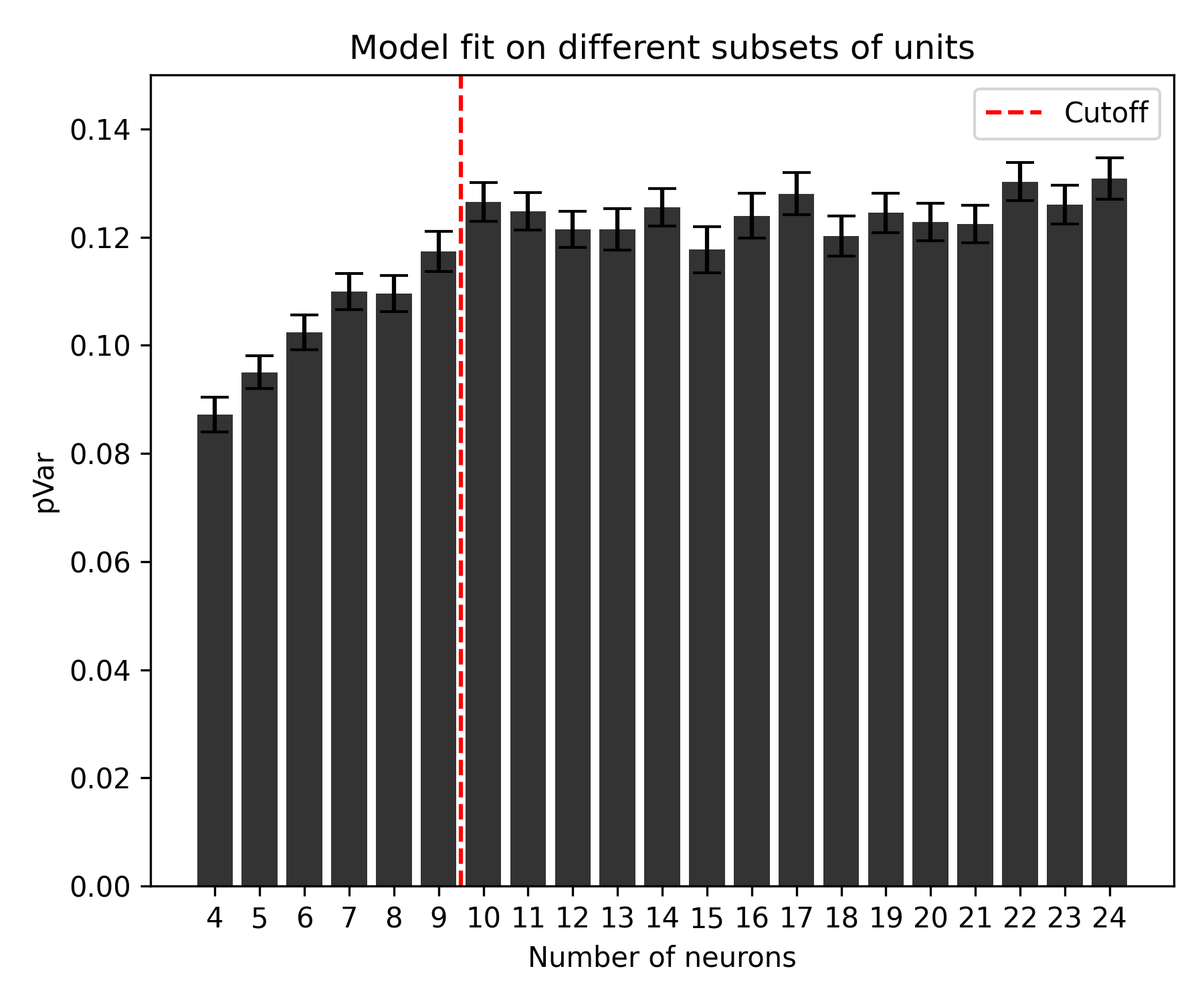


**Figure 5. Model goodness of fit as a function of the number of recorded neurons.** Systematic testing of partial variance (pVar) explained by CURBD models fitted on a subset of neurons from a sample dataset (500 resamplings per number of neurons) shows how pVar varies as a function of increasing number of neurons. We analyzed which bins were significantly different from the adjacent ones (where a bin indicates the number N of neurons included in the model). This analysis revealed that only two adjacent bins were statistically different from each other: the bins N=9 and N=10 (t-test, p = 0.018) and the bins N=13 and N=14 (t-test, p = 0.037). Moreover, to further explore whether the goodness of fit of the model showed a plateau after a certain number of neurons is included, we took the subset of neurons with the highest explained variance pVar (N=24) and compared all the other bins to it. This analysis showed us that all statistical comparisons from the subset N= 10 until N = 23 were not significantly different (Bonferroni corrected t-test, alpha = 0.01). All bins from N = 4 until N = 9 were significantly different from the N=24 bin (Bonferroni corrected t-test, p <0.001). All recording sessions with less than 10 neurons (red dashed line: cutoff) will therefore be excluded from further analyses.


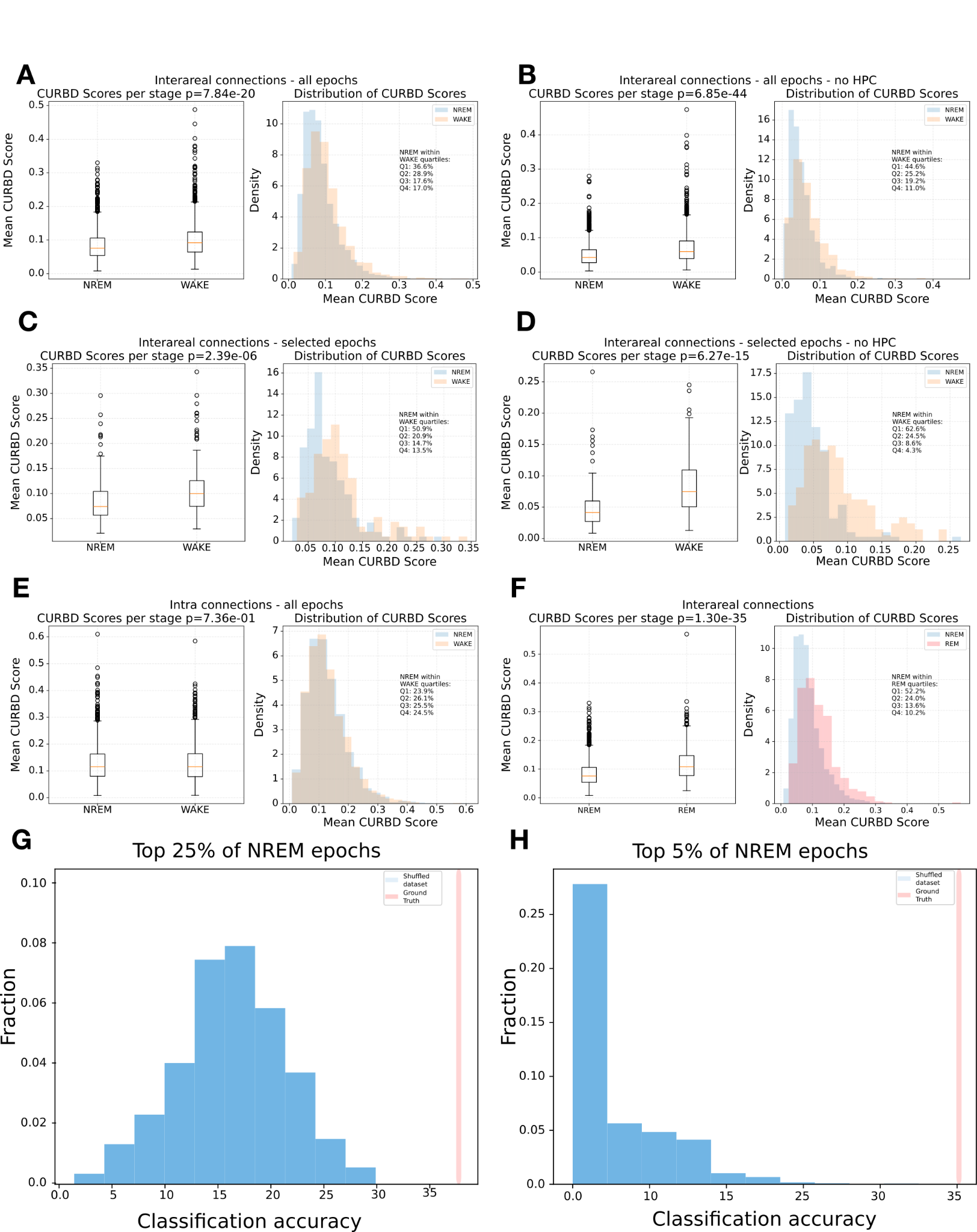


**Figure 6. Pilot results for a randomly selected recording session. A.** Left: Box plots showing the distribution of average inter-areal (aiFC) values for NREM vs. wakefulness epochs. Right: Histograms of the distribution of aiFC values for NREM vs wakefulness, with indicated the fraction of NREM epochs falling within each quartile of the distribution of aiFC values in wakefulness. **B.** Same as *A*, but excluding connections to and from the hippocampus. **C.** Same as *A,* but only for selected NREM epochs with the top 10% of SWA values and wakefulness epochs with the top 10% of detected motor activity. **D.** Same as *C*, but excluding connections to and from the hippocampus. **E.** Same as *A*, but for intra-areal connections. **F.** Same as *A*, but for NREM vs. REM. **G.** Classification accuracy for a classifier trained to discriminate high-aiFC NREM epochs from wakefulness epochs. Red: Ground-truth accuracy. Blue: Histogram of the accuracy obtained for classifiers trained on shuffled datasets. **H.** Same as *G*, but when considering NREM epochs with the top 5% of aiFC values.

|  | E | I |
| --- | --- | --- |
| C_m_ (pF) | 123.41 | 70.95 |
| gL (nS) | 2.47 | 9.49 |
| t_ref_ (ms) | 3 | 1.26 |
| V_rest_ (mV) | -80.97 | -82.35 |
| V_th_ (mV) | -40.53 | -56.32 |

**Table 1**: Parameters values for the two groups of cells. The values are taken from the Allen database (<https://portal.brain-map.org/explore/models/mv1-all-layers>) of cells in layer 2/3 of V1, as an example of a realistic network and without loss of generality of our method.

|  | | Target | |
| --- | --- | --- | --- |
|  |  | E | I |
| Source | E | 0.16 | 0.395 |
|  | I | 0.411 | 0.451 |

**Table 2**: **Within-area connection probability.** Probability of connections between populations in the same area. Sending population is indicated in the header of the first column of the table and receiving population is indicated in the header of the first row of the table. All values were taken from [(Billeh et al., 2020)](https://www.zotero.org/google-docs/?xOqxvN).

|  | | Target | |
| --- | --- | --- | --- |
|  |  | E | I |
| Source | E | 0.36 | 0.149 |
|  | I | 0.48 | 0.68 |

**Table 3**: **Connection strength within area.** Same as Table 2, but for intra-area connection strength
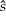
.

|  |  | Target | | | |
| --- | --- | --- | --- | --- | --- |
|  |  | E1 | E2 | E3 | E4 |
| Source | E1 | / | 6.93 10^-4 | 3.68 x 10^-5 | 7.16 10^-5 |
|  | E2 | 1.34 10^-3 | / | 3.17 x 10^-4 | 2.84 10^-4 |
|  | E3 | 6.37 10^-5 | 5.61 10^-4 | / | 9.33 x10^-45 |
|  | E4 | 1.29 10^-4 | 2.67 10^-4 | 1.47 10^-4 | / |

**Table 4**: Synaptic strength between excitatory neurons in the four different areas (E1: excitatory neurons in R1, etc.).

| Animal ID | Session type | BLA  (number of units) | PFC  (number of units) | A1  (number of units) | HPC  (number of units) | NREM  (number of 5s epochs) | REM  (number of 5s epochs) | WAKE (number of 5s epochs) |
| --- | --- | --- | --- | --- | --- | --- | --- | --- |
| r14 | Habituation | 7 | 7 | 16 | 1 | 1658 | 541 | 665 |
| r14 | Fear Conditioning | 6 | 9 | 14 | 3 | 1475 | 637 | 853 |
| r14 | Fear Test | 8 | 11 | 11 | 5 | 1261 | 661 | 1464 |
| r16 | Fear Conditioning | 6 | 6 | 4 | 5 | 2641 | 804 | 1169 |
| r20 | Habituation | 7 | 11 | 8 | 4 | 2556 | 772 | 1035 |
| r20 | Fear Conditioning | 4 | 9 | 10 | 4 | 2511 | 1290 | 2129 |
| r20 | Fear Test | 8 | 5 | 1 | 5 | 2065 | 752 | 1382 |

Table 5. Dataset description
